# Profiling of ocular surface microbiome and its ocular types in children and adolescents

**DOI:** 10.1038/s43856-026-01667-7

**Published:** 2026-06-08

**Authors:** Xiangtian Ling, Yuzhou Zhang, Christine H. T. Bui, Hei-Nga Chan, Yu Peng, Xiu Juan Zhang, Charlene C. Yim, Jennifer Wing-Ki Yau, Ka Wai Kam, Patrick Ip, Alvin L. Young, Kam Lun Hon, Clement C. Tham, Chi Pui Pang, Li Jia Chen, Jason C. Yam

**Affiliations:** 1 Department of Ophthalmology and Visual Sciences, The Chinese University of Hong Kong, Hong Kong SAR, China; 2 Department of Ophthalmology, The University of Hong Kong, Hong Kong SAR, China; 3 Hong Kong Eye Hospital, Kowloon, Hong Kong SAR China; 4 Department of Ophthalmology, Hong Kong Children Hospital, Hong Kong SAR, China; 5 Department of Paediatrics, The Chinese University of Hong Kong, Shatin, Hong Kong SAR China; 6 Department of Ophthalmology and Visual Sciences, Prince of Wales Hospital, Hong Kong SAR, China; 7 Department of Paediatrics and Adolescent Medicine, LKS Faculty of Medicine, The University of Hong Kong, Hong Kong SAR, China; 8 Hong Kong Hub of Paediatric Excellence, The Chinese University of Hong Kong, Hong Kong SAR, China

**Keywords:** Computational biology and bioinformatics, Conjunctival diseases

## Abstract

**Background:**

The ocular surface microbiome is increasingly recognized for its role in eye health, but the complexity of its composition complicates personalized characterization. This study aims to define the ocular surface microbiome profile and identify distinct microbial community clusters, or Ocular Types, in a population of children and adolescents.

**Methods:**

In this population-based, cross-sectional study, conjunctival swabs from children and adolescents were processed with 16S ribosomal RNA gene amplicon sequencing. Microbial clusters were determined using a partitioning around medoids clustering approach, validated through internal cross-comparison and external datasets. Functional profiles of the clusters were predicted from the sequencing data.

**Results:**

Here we show that in 1246 samples from 1006 individuals aged 3 to 18 years, the predominant bacterial phyla are Proteobacteria (34.1%), Firmicutes (37.4%), and Actinobacteria (25.2%), with *Staphylococcus*, *Corynebacterium*, and *Streptococcus* as core microbiota. Five distinct Ocular Types are identified, characterized by the dominance of *Staphylococcus*, *Corynebacterium*, *Streptococcus*, uncultured *Neisseriaceae*, or *Escherichia-Shigella*. These Ocular Types demonstrate associations with host factors including age, ethnicity and ocular parameter, but not with sex. Functional prediction reveals considerable overlap in the metabolic pathways among the Ocular Types.

**Conclusions:**

This study characterizes five distinct ocular surface microbiome types in pediatric population and establishes their association with host factors. These findings provide a structured framework for investigating microbial community dynamics and their potential implications for ocular health.

## Plain language summary

This study explored the types of bacteria living on the eye surface in 1006 children aged 3–18 living in Hong Kong. Using genetic testing on swab samples, we identified five main types of bacterial communities, each dominated by different common bacteria. These community types were associated with a child’s age, ethnic background and ocular health but not their sex. Interestingly, while the bacterial members differed, their predicted functions were similar. Our findings provide a clear framework for understanding how the eye’s microscopic ecosystem varies between children and may influence eye health.

## Abstract (web summary)

Ling, Zhang et al., profile the ocular surface microbiome in a paediatric cohort, identifying five distinct community types. These communities link to ethnicity and ocular surface disease index, regardless of sex and reveal conserved metabolic pathways across different microbial structures

## Introduction

Ocular surface microbiome (OSM) refers to the community of microorganisms and their genetic materials on the surface of the human eye^1,2^. A well-balanced OSM contributes to ocular surface homeostasis, maintenance of the immune system, and defense against pathogens. An unbalanced OSM could lead to pathogenic microbial overgrowth and the over-production of metabolites with inflammatory and/or disease-contributing properties, thus provoking or perpetuating the development of ocular diseases. Variations in OSM have been implicated in a range of ocular conditions^2^, from dry eye syndrome^3–8^, allergic rhinoconjunctivitis^9^, and high myopia^10^, to more severe afflictions including trachoma^11,12^, keratitis^13–15^, diabetic retinopathy^16–18^, and conjunctival mucosa-assisted lymphoid tissue lymphoma^19^.

Eye health is profoundly important during childhood, a formative period for sensory development. Most current studies focus on adults’ OSM, which significantly differs from that of children^11,20^. It is necessary to characterize the compositions of OSM during this developmental period and understand its role in ocular health.

There are various microbes on the ocular surface, and variations in the bacterial compositions of the microbes have been identified across the world. These variations have hindered the establishment of a definitive consensus on the core microbiota^2,21^. Furthermore, the complexity of these microbial compositions poses challenges in characterizing the OSM accurately for individuals or specific populations. Consequently, the development of targeted therapies and preventative probiotics is impeded. To address this, a more structured framework for classification is needed.

In this study, we characterize the ocular surface microbiome profile in a large cohort of children and adolescents. Using a clustering approach, we identify five distinct microbial community types, Ocular Types, and analyze their associations with host factors. This work provides a structured framework for understanding microbial community dynamics and establishes a foundation for exploring their implications for ocular health.

## Methods

### Study population

This cross-sectional analysis enrolled participants from the Hong Kong Children Eye Study (HKCES)^22,23^, an ongoing, multi-ethnic, population-based cohort investigating ocular health in schoolchildren. Participant recruitment employed a stratified, clustered random sampling strategy across seven geographical districts in Hong Kong. Enrolled children underwent comprehensive ophthalmic examinations physical examinations. Ophthalmic examinations included cycloplegic autorefraction, axial length, central corneal thickness measurement and other ocular biometry. Physical examination included the measurement of height and weight for the calculation of body mass index (BMI). Dry‑eye symptoms were assessed using Ocular Surface Disease Index (OSDI) questionnaire^24^. One question (Q7, Driving at night) (Q7, “Driving at night”) was deemed irrelevant to the pediatric population and was omitted^25^. The final score was calculated using the standard formula (sum of scores × 25 / number of questions answered) to account for the omitted item. Supplemental assistance was provided to children who required support in completing the questionnaire. Standard thresholds were applied (0–12 normal, 13–22 mild, 23–32 moderate, 33–100 severe).

All participants of HKCES from May 2022 to December 2023 aged 3-18 years were invited to join this study. Exclusion criteria consisted of a history of ocular trauma, congenital ocular malformations, inability to complete the examination protocol, and the use of topical eye drops or contact lenses within one week prior to sampling. The study procedure was performed in accordance with the Declaration of Helsinki. The study protocol was approved by the Ethics Committee Board of the Chinese University of Hong Kong (approval No. 2015.033). Written informed consent was obtained from the legal guardians, and verbal consent was obtained from the children.

### Sample collection

Conjunctival samples were obtained from participants, with collection from one or both eyes to enable bilateral comparison. Each eye received a drop of sterile proxymetacaine hydrochloride (ProVain-POS Eye Drops 0.5% w/v). Two minutes after topical anaesthesia, a sterile individually packaged Puritan flocked swab (Catalog No. 25-3000-H E30) was used to swab the inferior conjunctival fornix by gently rolling from the nasal to temporal sides and then back for at least 3 rounds. Swabs were immediately stored in sterile tubes, kept on ice, and transferred to a -80 °C freezer within two hours. To avoid external contamination, special care was taken to prevent the swab from contacting other ocular structures. Swabs with or without proxymetacaine hydrochloride eye drops were obtained as environmental controls from the air of the sample collection room.

### DNA extraction, PCR amplification, and 16S rRNA high-throughput sequencing

The DNeasy PowerSoil Pro Kit (Qiagen) was used to extract DNA from the swab samples within two weeks, following the manufacturer’s guidelines. DNA quantity was measured by Qubit 4 Fluorometer (Thermo Fisher). PCR amplification of V3-V4 region of bacterial 16S rRNA gene was performed with 341 F forward primer (CCTACGGGNGGCWGCAG) and 805 R reverse primer (GACTACHVGGGTATCTAATCC)^26^ following the 16S Illumina Demonstrated Library Prep Guide (# 15044223 Rev. B). Twenty-five amplification cycles were performed. High-throughput sequencing was performed on an Illumina MiSeq platform.

### Processing of sequencing data

Processed with the Qiime2 (2023.05) suite^27^, our sequencing data underwent denoising by DADA2^28^, generating amplicon sequence variant (ASV) tables and their corresponding sequences. Taxonomic classification drew upon q2-feature-classifier, custom-trained with our primer data, and referenced against the SILVA 138 database^29^. We applied the R software for the filtration of rare ASVs, those singular to one sample or with an occurrence below ten counts throughout all samples, alongside the *Decontam* package for contamination removal with prevalence-based contaminant identification (threshold=0.5)^30^. Samples presenting less than 10,000 ASVs were excluded. Core microbiota was defined by a detection level of 1% and a prevalence of 50%^31,32^.

### Microbial community clustering

The Calinski-Harabasz (CH) index and instantiated Partitioning Around Medoids (PAM)-based clustering, alongside Jensen-Shannon divergence metrics, were applied for determining the optimal number of microbiome clusters^33^. In order to understand how the sample size would influence the optimal number of clusters, we randomly selected specific quantities of samples (10, 20, 50, 100, 150, 200, 300, 400, 500, 600, 700, 800, 1000) and repeated the process 1000 times. We then identified the best cluster number using the peak value of the CH index and summarized the optimal number for each specific quantity (sample size). To verify the reliability of our clustering outcome (Ocular Type), we divided our dataset randomly into two subsets, allocating 60% and 40% to each, and assessed the consistency between the results of these two subsets. Additionally, we further validated our findings by comparing them with five external available 16S datasets^5,7,9,16,34^.

### Statistics and reproducibility

All statistical analyses were performed in R (version 4.1.3). Differentially abundant taxa across the defined ocular types were identified using Linear discriminant analysis (LDA) Effect Size (LEfSe). The metabolic pathway abundance was predicted by PICRUSt2^35^ and compared by LEfSe. Correlations between demographic data, ocular parameters, and ocular types (categorical) were made using Point-Biserial Correlation Coefficients, with the *P* values adjusted for multiple comparisons using false discovery rate (FDR) correction. Alpha and beta diversity metrics were calculated using the *vegan* package. The sample size (*n* = 1246 samples) was determined by the availability of qualified samples meeting the sequencing depth threshold during the study period. Replicate analysis, in the context of clustering validation, was defined as the repeated PAM clustering performed on 1000 random subsets of the full dataset. No other experimental replicates were conducted, as this was an observational, sequencing-based study.

### Ethics approval

 The study procedure was performed in accordance with the Declaration of Helsinki. The study protocol was approved by the Ethics Committee Board of the Chinese University of Hong Kong (approval No. 2015.033). Written informed consent was obtained from the legal guardians, and verbal consent was obtained from the children.

## Results

### Overview of the microbiome in children

A total of 1283 conjunctival samples and 32 environmental controls were collected and sequenced. After data decontamination and ASVs filtering, 1246 samples from 1006 participants included (240 of whom contributed samples from both eyes) were for further analysis. The mean age for the included 1006 participants was 10.2 (SD: 2.6, ranged 3-18) years, with 53.7% are boys. This child cohort consisted of 795 Chinese (79%), 83 Pakistani (8%), 65 Nepalese (6%), 24 Indian (2%), and 39 from other ethnicities (4%). ASVs per sample ranged from 10,158 to 93,551 counts, with an average of 37,995 ± 15,075.

**Fig. 1:**
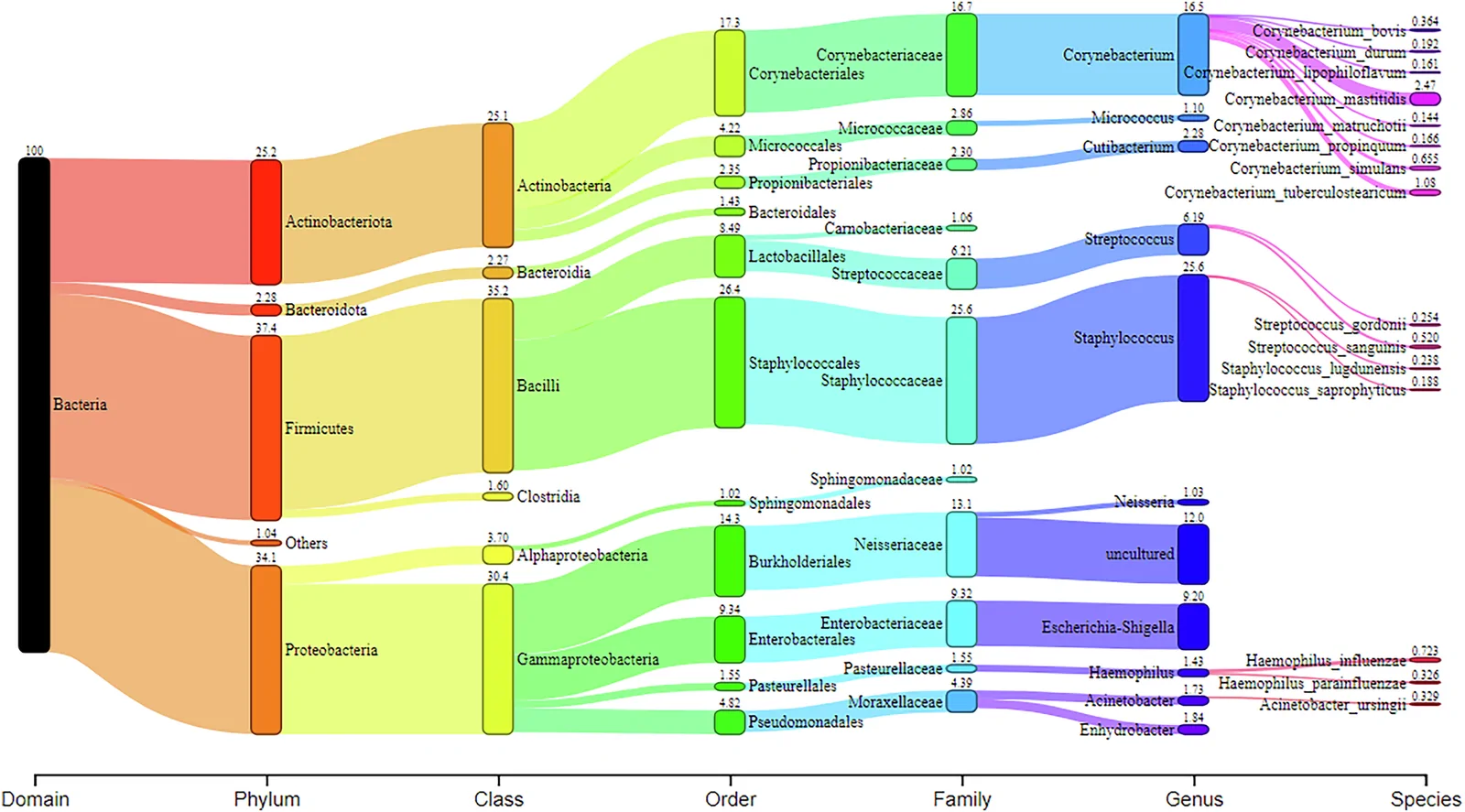
Sankey diagram of the taxonomic composition of bacterial communities in ocular surface microbiome (OSM) samples. Taxa are shown hierarchically from left to right across Domain, Phylum, Class, Order, Family, Genus, and Species. Coloured flows indicate taxonomic relationships. Numeric values above nodes indicate relative abundance percentages.

The ocular surface microbiome was predominated by the phyla Firmicutes (relative abundance: 37.4%), Proteobacteria (34.1%) and Actinobacteriota (25.2%), which collectively constituted over 95% of the bacterial composition (Fig. 1). At the genus level, the most abundant ones included *Staphylococcus* (25.6%), *Corynebacterium* (16.5%), uncultured *Neisseriaceae* (12.0%), *Escherichia-Shigella* (9.2%) and *Streptococcus* (6.19%; Fig. 1 and Supplementary Fig. 1a). The most prevalent identifiable species was *Corynebacterium mastitidis*, constituting 2.47% of the total bacteria. However, imposing a detection level of 1% and a prevalence cutoff of 50%, only three core microbiota emerged, namely *Staphylococcus, Corynebacterium* and *Streptococcus* (Supplementary Fig. 1B).

Paired samples from the right and left eyes of the same participant (n = 240) showed no significant difference in microbial composition. (Supplementary Fig. 2). Furthermore, the beta diversity (Bray-Curtis dissimilarity) between samples from the same individual was significantly lower than that between samples from different individuals (Mann-Whitney U test, *P* < 0.001), indicating consistent personal microbial profiles and supporting the robustness of data (Supplementary Fig. 2D).

### Determination of ocular types

**Fig. 2:**
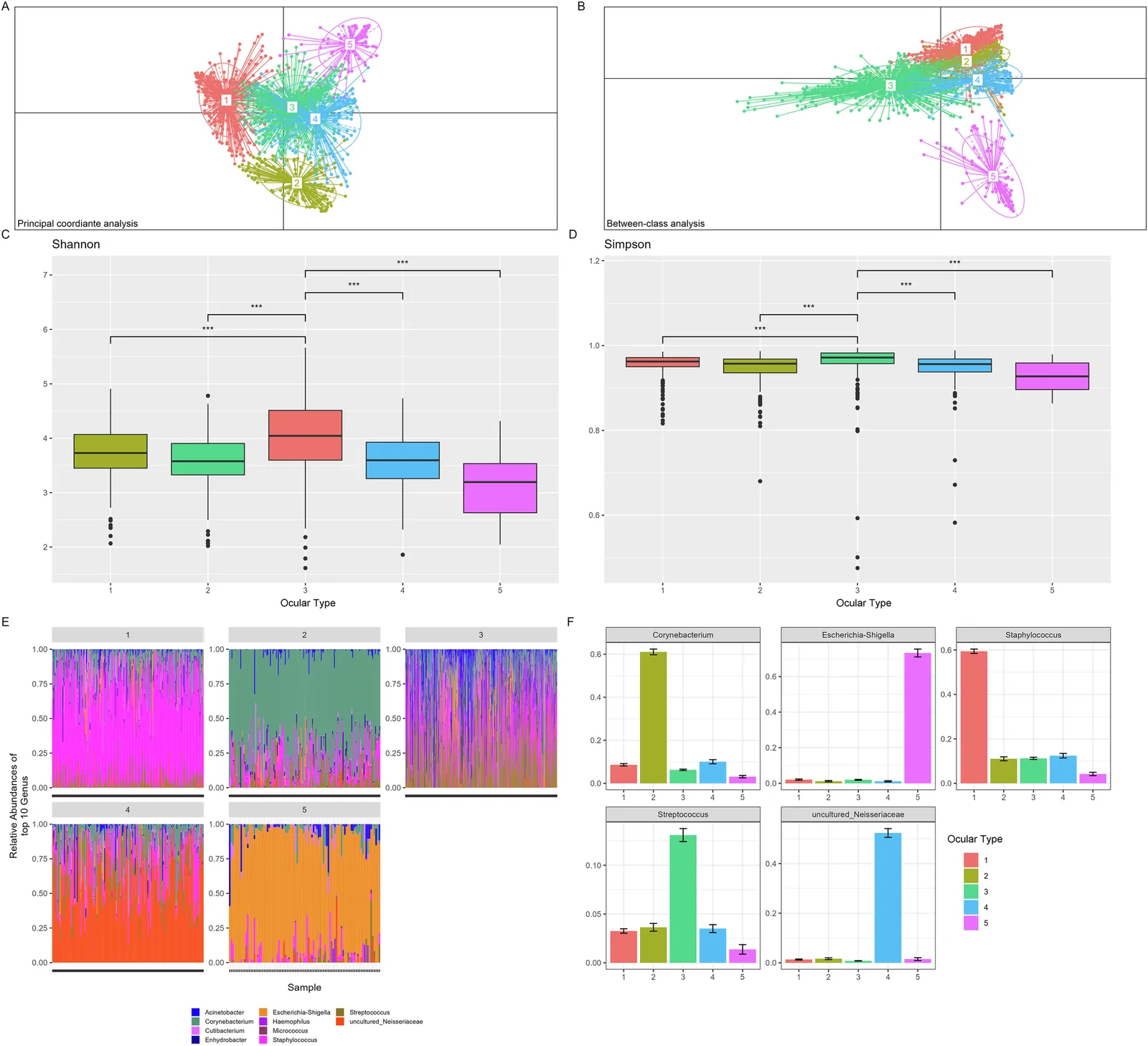
Identification of five distinct ocular types within the OSM of Hong Kong children. **A** Between-class analysis revealing differences among the five ocular types. **B** PCoA highlighting the separation of the five ocular types. **C** Boxplots comparing Shannon diversity indices across the five ocular types, with significant differences marked by asterisks where applicable. *Whiskers* represent the range within 1.5 × inter-quartile range (IQR), and the points beyond this range are plotted as outliers. **D** Boxplots comparing Simpson diversity indices across the five ocular types, with significant differences marked by asterisks where applicable. *Whiskers* represent the range within 1.5 ×IQR, and the points beyond this range are plotted as outliers. **E** Bar graph indicating the relative abundance of bacterial composition at genus level per sample within an ocular type. **F** Bar graph showing the relative abundance of distinct genera (Staphylococcus, Corynebacterium, Streptococcus, uncultured Neisseriaceae, and Escherichia-Shigella) within each ocular type. Data represent mean ± standard error of the mean (SEM). (***p < 0.001). The corresponding number of Ocular Type 1-5 is *n* = 322, *n* = 173, *n* = 481, *n* = 178, *n* = 92.

The optimal cluster number of microbial clusters was determined to be five, based on the peak CH index (Supplementary Fig. 3). Between-class analysis (BCA) and principal coordinate analysis showed the structural difference among these five ocular types (Fig. 2A, B). Ocular Types 1, 2, 4 and 5 demonstrated clear segregation, albeit with slight overlaps with Ocular Type 3. The silhouette coefficient, affirming the delineation, indicated satisfactory coherence within Ocular Types 1, 2, 4 and 5, whereas Ocular Type 3 exhibited instability in nearly half its samples, as evidenced by negative silhouette coefficients (Supplementary Fig. 4). With respect to alpha diversities, Ocular Type 3 surpassed others in Shannon and Simpson indexes (Fig. 2C, D). The primary genera that defined each ocular type were found to be varied, with Ocular Types 1, 2, 4 and 5 being predominated by specific taxa, while Ocular Type 3 displayed a more heterogeneous composition (Fig. 2E). Discriminant markers among the ocular types were identified via LEfSe (Supplementary Fig. 5, LDA score >4, *P* < 0.01 for Kruskal-Wallis Test), revealing dominant genera including *Staphylococcus* (Ocular Type 1), *Corynebacterium* (Ocular Type 2), *Streptococcus* (Ocular Type 3), *uncultured Neisseriaceae* (Ocular Type 4), and *Escherichia-Shigella* (Ocular Type 5; Fig. 2F).

### Validation of ocular types

Internal validation through a 60/40 random split of the dataset consistently recovered the same five ocular types in both subsets (Supplementary Tables 1—2 and Supplementary Figs. 6—13). External validation using five independent public datasets confirmed that the microbial signatures defining our ocular types are recognizable across diverse populations. The first dataset was from Yau’s study on allergic rhinoconjunctivitis^9^, which included samples from 24 children in Hong Kong. These samples could be clustered into 3 ocular types (Supplementary Fig. 14), which were predominated by *Staphylococcus*, *Streptococcus*, and an unknown genus from the *Neisseriaceae* family (Supplementary Table 3, Supplementary Figs. 15 and 16). The second dataset was from Ozkan’s study on dry eye in Australian population^5^. In this dataset, 282 samples were stratified into three ocular types (Supplementary Fig. 17), which were represented by *Staphylococcus*, *Corynebacteria* and *QFNR01* (an uncultured genus in the *Neisseriaceae* family; Supplementary Table 4, Supplementary Figs. 18 and 19). Moreover, three more datasets in Chinses adults (Dong’s dataset on MGD, Song’s dataset on Sjögren’s Syndrome, Zhu’s dataset on diabetes) identified two or three ocular types as their clustering results. Even though these datasets yielded different outcomes, all the identified ocular types fit into the five ocular types that we had found in our present study (Supplementary Figs. 20–25).

### Associations of ocular types with demographic and ocular health

**Fig. 3:**
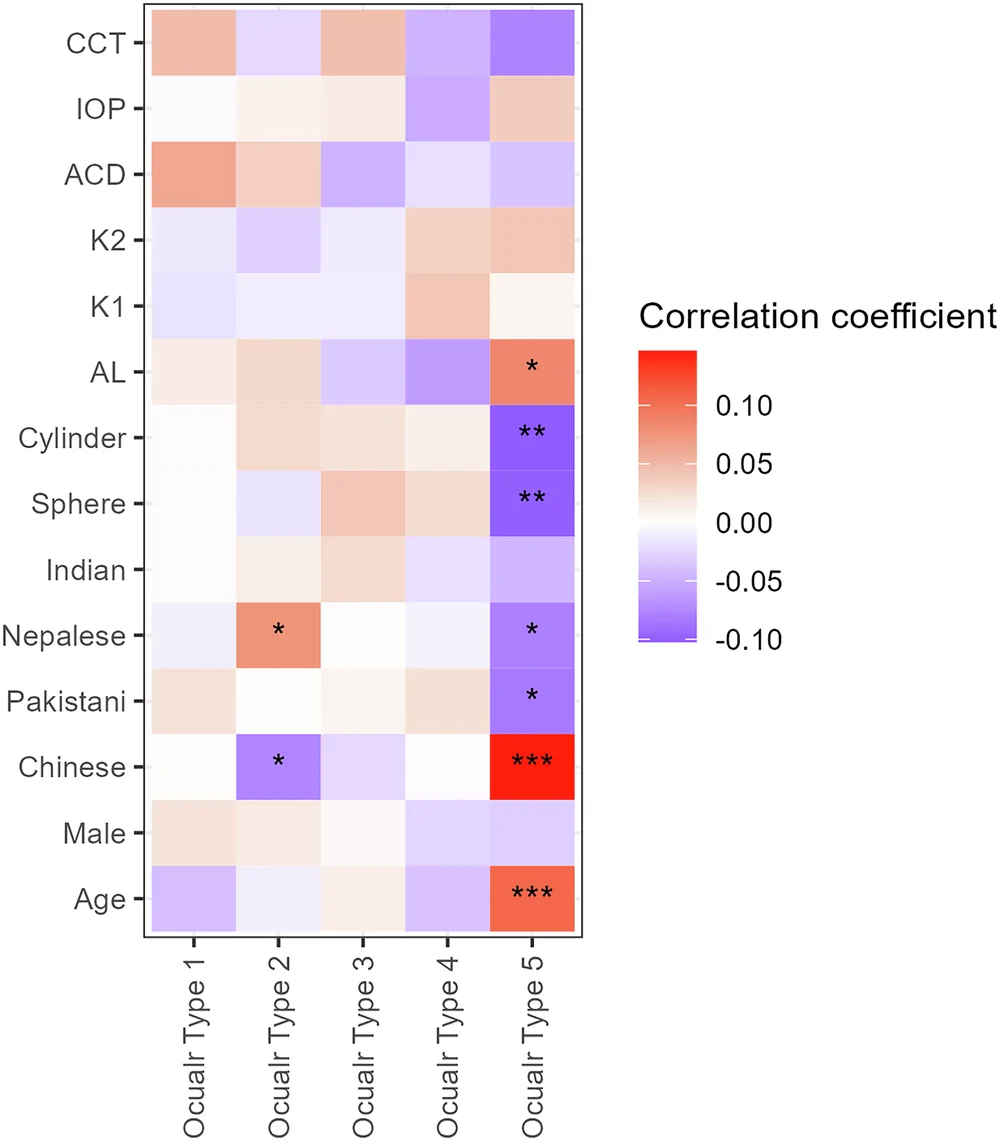
Correlation between ocular types with demographic data, ocular biometrics and OSM diversity. Heatmap summarizing the association between ocular types with demographic factors (age, sex, ethnicity) and the various ocular parameter, with colors indicating the correlation coefficients. AL Axial Length, ACD Anterior Chamber Depth, K1 Keratometry Reading 1, K2 Keratometry Reading 2, CCT Central Corneal Thickness, IOP Intraocular Pressure, with significance denoted by (*p < 0.05, **p < 0.01, ***p < 0.001). *P* values were adjusted with false discovery rate (FDR) correction.

Correlation analysis between microbiome clusters, demographic characteristics and ocular measurements revealed cluster-specific correlations. The demographic data of each ocular type was shown in Table 1. Remarkably, only Ocular Type 5 displayed a significant correlation with age (Fig. 3). No ocular types were significantly associated with sex. On the other hand, ethnic backgrounds manifested distinct associations. All samples of Ocular Type 5 were from Chinese children, whereas Nepalese children showed a correlation with Ocular Type 2. In terms of ocular parameters, Ocular Type 5 showed significant correlations with parameters for spherical equivalent and axial length (Fig. 3).

**Table 1:** Demographic data of different ocular types

Ocular Type	1	2	3	4	5
Representative genus	Staphylococcus	Corynebacterium	Streptococcus	uncultured Neisseriaceae	Escherichia-Shigella
Percentages of samples	25.8%	13.9%	38.6%	14.3%	7.4%
Age	10.1 ± 2.6	10.2 ± 2.6	10.3 ± 2.7	10.1 ± 2.4	11.3 ± 2.4
Male %	55.6%	55.8%	54.1%	50.0%	48.4%
Chinese %	78.9%	71.1%	77.5%	78.7%	100%

Analysis of the external dataset from the study on dry eye in an Australian population found an association between the ocular type characterized by uncultured *Neisseriaceae* and dry eye disease (Supplementary Table 5). Consistent with this finding, analysis of the OSDI scores within our own cohort revealed a difference in the proportion of participants with dry eye across the five ocular types (Kruskal-Wallis rank sum test, *P* = 0.03). Notably, the lowest prevalence of non-dry eye symptoms was observed in Ocular Type 4 (uncultured *Neisseriaceae*-dominant).

### The functional prediction of ocular types

**Fig. 4:**
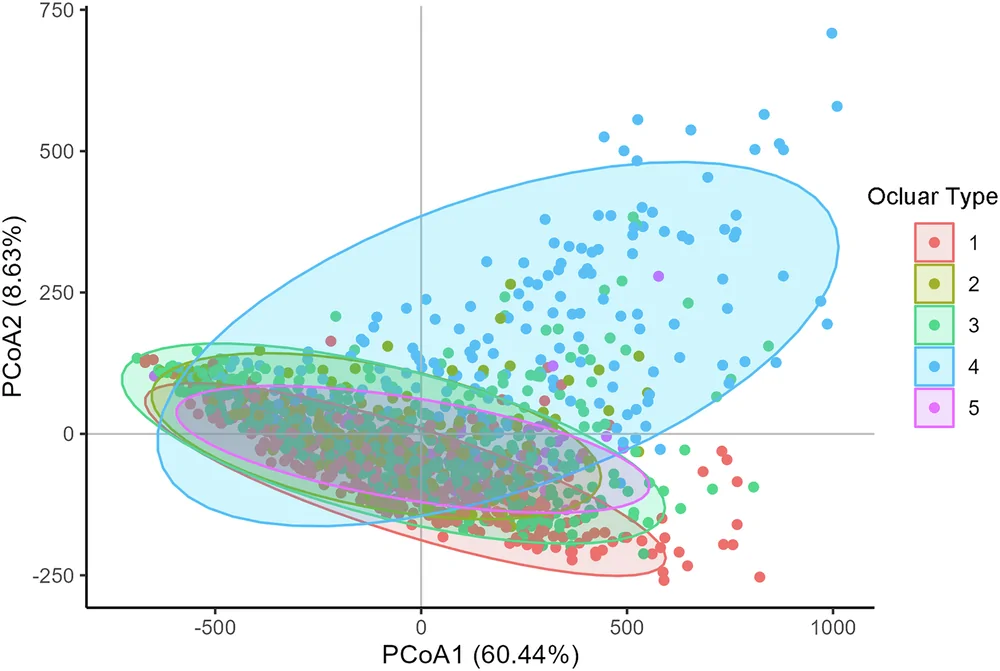
Principal coordinate analysis illustrating the distribution of Metacyc pathways. Each filled point represents one sample and is coloured by ocular type. The PCoA1 axis explains 60.44% of the variance and the PCoA2 axis explains 8.63%. Semi‑transparent shaded ellipses, outlined in the corresponding group colour, represent 95% confidence intervals for each ocular type, illustrating within-group dispersion and overlap among groups in ordination space.

Functional comparison across the ocular types, processed by Metacyc pathway^36^ predictions via PICRUST2, showed considerable overlaps among the ocular types on principal coordinates analysis (PCoA, Fig. 4). LEfSe identified distinct pathways among Ocular Types (Supplementary Fig. 26): acetylene degradation (anaerobic), ppGpp (guanosine tetraphosphate and pentaphosphate) metabolism, glycogen biosynthesis I, aerobic respiration and formaldehyde assimilation II.

## Discussion

In this study, we characterized the ocular surface microbiome in a multiethnic cohort of Hong Kong children and adolescents aged 3 to 18 years, using the largest OSM dataset to date. We identified the abundant bacteria present and discover five distinct ocular types in Hong Kong children with ethnic differences. This finding enriches the growing understanding of the complexities of the OSM and simplifies these high-dimension data, providing an important reference for future study. Furthermore, our exploration into the functional pathways hinted at the potential significance of the ocular surface microbiome in ocular health.

When comparing our findings with external datasets, we found that the number of clusters identified by external datasets were less than our delineated five types. Notably, the size of the sample pool emerged as an important factor influencing clustering outcomes. Given that we have a substantial dataset comprising 1246 samples, we determined in this study that the optimal cluster number was five. However, simulations with reduced sample sizes showcased the variability in the ideal cluster number, indicating a stronger convergence towards five clusters as sample numbers exceeded 300 (Supplementary Figs. 27 and 28). With sample sizes surpassing 1000 in our dataset, it became apparent why all five ocular types were not universally recognized by external datasets. Another key consideration for the absence of certain ocular types in external datasets could be due to the rarity of specific types, potentially limited to distinct populations. For example, Ocular Type 5, distinguished by *Escherichia-Shigella*, was only found in Chinese children, illustrating the nuanced distribution of ocular microbiome profiles across populations.

Interestingly, the ocular types appeared to be continuous rather than strictly discrete entities. This continuity was exemplified by Ocular Type 3, which was characterized by a diverse array of bacterial compositions, including more than 10 marker genera that were rare in the other clusters. This diversity might confer a greater ecological stability and resilience against pathogens, drawing on the principles of ecosystem theory^37^. The potential link between ocular microbiome clusters and diseases also merits further exploration, as our analysis of external datasets hinted at the microbiome’s role in ocular diseases.

We observed cluster-specific demographic and ocular associations. Notably, Ocular Type 5 (*Escherichia-Shigella* dominant) occurred only among Chinese children and showed a positive association with age within this pediatric cohort. In an independent Hong Kong adult cohort (n = 130, ages 28–61 years), Ocluar Type 5 was not detected; all samples mapped to Ocular Types 1–4, and age was positively associated with Ocular Type 2 (*Corynebacterium*-dominant) (Supplementary Table 6). Taken together, these findings suggest that age might relate to ocular community state. While age is a well-recognized determinant of gut and skin microbiomes^38^, its role in shaping the pediatric OSM need confirmation in longitudinal cohorts spanning a broader age range.

Despite links between nutrition and microbial community structure in the gut^39^, BMI did not differ across Ocular Types in our cohort (*P* = 0.22, ANOVA test, Supplementary Table 7). This is biologically plausible given the limited direct nutritional exposure of the conjunctival surface, although systemic factors cannot be excluded. We also find ethnic associations that all Ocular Type 5 samples occurred in Chinese children, while Nepalese children were enriched for Ocular Type 2, pointing to possible contributions from genetic background, environment, and lifestyle. Previous research on ethnic variation in the ocular microbial composition is limited, with only one culture-based study reporting differences between two Chinese ethnic groups^40^. Directly comparing microbial compositions across distinct geographical regions is challenging due to geography itself being a significant confounder^41^. Therefore, a well-designed, multi-ethnic study is required to validate these ethnicity-related findings, which may provide critical insights into disease risk and differential treatment responses.

Predicted functional profiling showed that, despite compositional differences, Ocular Types shared a large overlap in pathway space on PCoA, points towards a potential “functional core”^42^. Defining such core functions may guide the design of microbiome‑targeted interventions to restore “healthy microbiome” in dysbiotic states^42,43^. Even so, several distinguishing pathways, including enrichment of ppGpp metabolism, glycogen biosynthesis and aerobic respiration were differentially enriched across Ocular Types, suggesting community-specific metabolic priorities. Such differences may have implications for host–microbe interactions, particularly in relation to stress adaptation, persistence strategies, carbon storage, and energy utilization.

There are several limitations in this study. First, the cross-sectional design restricts our ability to track changes in ocular types over time, preventing the assessment of age-related effects. Second, the reliance on 16S rRNA sequencing, with its species-level resolution limitations and the potential bias induced by primers targeting specific regions, may have affected the accuracy of our findings. Shotgun metagenomics, metatranscriptomics with host immune profiling would enable provide higher-resolution taxonomic (including strain-level) and functional insights. Third, missing covariates including general or ocular hygiene practices and environmental exposures limit the granularity of association analyses, which will require more comprehensive data collection in future studies to investigate their impact on ocular microbial variation.

## Conclusions

In conclusion, our study characterized the complex compositions and diversities of ocular surface microbiome in Hong Kong children. Our work on ocular types enriched the growing understanding of the microbial community on the ocular surface, and its associations with host factor including age and ethnicity. This investigation has also emphasized the necessity for future study, particularly the roles of different ocular types in ocular health and their associations with ocular diseases.

## Supplementary information

Transparent Peer Review file

Supplementary materials

## Data Availability

The sequencing data generated in this study have been deposited in the NCBI Sequence Read Archive database under accession code PRJNA1415892. The publicly available raw data were downloaded through retrieved accession numbers including PRJNA564695 and PRJNA802065 from cited papers. Source data for Figs. 1 - 4 can be accessed via the Zenodo repository^44^.

## Code availability

All software used is from publicly available source. Codes used for the microbiome analyses are available via GitHub at https://github.com/ficion1/Profiling-of-OSM-in-Hong-Kong-Children.
