# Supplementary material for: Profiling of ocular surface microbiome and its ocular types in children and adolescents
Source: Commun Med (Lond). 2026 Jun 8;6:487. doi: 10.1038/s43856-026-01667-7 (PMC13582937; doi:10.1038/s43856-026-01667-7)
Supplement: Supplementary file 1 [file 43856_2026_1667_MOESM1_ESM.pdf]

nature portfolio

# Peer Review File

# Profiling of ocular surface microbiome and its ocular types in children and adolescents

Corresponding Author: Professor Jason Yam

**This file contains all reviewer reports in order by version, followed by all author rebuttals in order by version.**

Version 0:

Reviewer comments:

Reviewer #1

(Remarks to the Author)
This manuscript reports a robust, population-based cross-sectional study that characterizes the ocular surface microbiome (OSM) in 1,006 children and adolescents in Hong Kong using 16S rRNA sequencing. The authors successfully identify five distinct microbial clusters ("ocular types") through PAM-based clustering and investigate their associations with demographic factors and ocular parameters, along with functional pathway predictions. However, the proposed ocular "types" would benefit from longitudinal validation and additional functional correlations, such as immune profiling or clinical outcome data, to strengthen their relevance in clinical practice.

Reviewer #2

(Remarks to the Author)
Please mention the IEC approval no. Please clearly explain the inclusion criteria. Please mention the main parameters considered for the comprehensive ophthalmic and physical examinations. Were the kids selected randomly or consecutive enrolment? The age-group considered, and the subgroups based on age?
How was it selected if one or both eyes of patients will be sampled? Was a smaller size paediatric swab used for the collection?
Was the nutritional status of the kids and hygiene (general and ocular) taken into consideration during analysis as this might also alter the surface microbiome?
Uncultured Neisseriaceae was known to be associated with dry eye. Did these kids in this cohort with this organism have any dry eye?
L222-230: For the functional prediction of ocular types, please explain the significance of finding these associated metabolic pathways.
Discussion
The authors clearly explained here that the purpose of this study was to classify the 5 types of OSM in children in Hongkong with different ethnicities, so that it would serve as a reference for future studies. But it would be good if they did some more correlation of the data to show how different OSM was related to different ethnicities' ocular surface diseases in that age group from any previous publications and how it was related to nutritional status of patients etc.
Overall, the work, though innovative, needs reanalysis and further correlation studies and literature review to make the findings more robust and impactful.

Reviewer #3

(Remarks to the Author)
This study aimed to define ocular types in a cohort of children aged between 3 and 18 years. It profits from a large sample size but suffers from a small age range to do adequate statement about age-related findings. For details, see the following concerns.

Major concerns:
1) The age range is too small to make a statement about age-related ocular types. To assess this, the cohort should include participants of a larger age range.

2) Line 176: The absence of significant differences between the right and left eye does not necessarily implicate consistent and robust data. Are the data between the left and right eye more similar that the data of eyes from different participants?

Minor concerns:
1) line 69: The term «microbiome» refers to the microbes and their genetic entities in a defined niche. Please adapt the term accordingly.

2) Missing information:
– inclusion criteria: Was intake of antibiotics in the last few months not an exclusion criteria? If not, was intake of antibiotics recorded during the recruitment process?
– How many PCR cycles have been performed (line 120)?
– Which method was used in Decontam for removal of potential contaminants (line 131)?
– line 150: why did you use LEfSe for taxonomic analysis and Maaslin2 for pathway analysis? Which parameter did you use for both of them?
– Line: 207: How were dry eyes assessed?

3) line 161: the cohort is not balanced between the different ethnicities.

4) line 173: is the term "core taxa" according to your definition of a detection level of 1% and a prevlance of 50%? If yes, is this definition in accordance with previously published ocular surface microbiome data?

Version 1:

Reviewer comments:

Reviewer #1

(Remarks to the Author)
The manuscript has been thoroughly revised to address all reviewers' comments and is now ready for publication.

Reviewer #3

(Remarks to the Author)
Thank you for providing the revised manuscript that improved a lot, mainly by adding the older age cohort. However, I still have some concern that have to be considered.

1) It remains unclear why different statistical tools were applied for the taxonomic and functional analyses, specifically the use of LEfSe for taxonomic composition and MaAsLin2 for pathway analysis. This choice raises concerns regarding methodological consistency and comparability of results within the same study.
MaAsLin2 is specifically designed to detect associations between microbial features and metadata variables within a multivariable modeling framework. Importantly, it allows adjustment for potential confounders and supports the inclusion of random effects, thereby accounting for complex study designs and reducing the risk of biased associations. In contrast, LEfSe is primarily a univariate biomarker discovery tool and does not provide a framework for adjusting for confounding variables.
Given these fundamental differences in statistical approach, the rationale for applying LEfSe to taxonomic data while using MaAsLin2 for pathway analysis requires clearer justification. If confounding factors were considered relevant for the pathway analysis, they should arguably also have been incorporated into the taxonomic analysis to ensure methodological coherence and internal consistency of the findings.
Furthermore, the manuscript should provide a detailed description of the MaAsLin2 model specifications. Please explicitly state:
Which variables were included as fixed effects?
Whether random effects were incorporated, and if so, which ones?
Providing these details is essential for transparency, reproducibility, and proper interpretation of the reported associations.

2) The use of the OSDI questionnaire in the present study requires further justification. The OSDI was originally developed and validated for adult populations and has not been specifically validated for use in children. Consequently, its applicability and validity in a pediatric cohort are questionable.Several items included in the questionnaire may not be age-appropriate and could compromise the reliability of the collected data. For example, questions referring to "difficulty driving at night" are clearly not applicable to children. In addition, younger children may have limited ability to accurately self-assess and quantify subjective ocular symptoms, further affecting the validity of the responses.Given these limitations, the authors should clarify whether a modified or adapted version of the OSDI was used and how age-appropriateness was ensured. Alternatively, the use of a pediatric-validated instrument would be more methodologically sound.

3) If the term "core taxa" is based on already published literature, please add the corresponding literature (Morandi et al.). It may be better to use "core microbiota" or "core microbiome" instead of "core taxa".

Version 2:

Reviewer comments:

Reviewer #3

(Remarks to the Author)
Thank you for providing the revised and updated manuscript in response to the reviewers' comments. The revisions have improved the overall quality of the manuscript. I have no further concerns and propose to accept the manuscript for publication.

**Open Access** This Peer Review File is licensed under a Creative Commons Attribution 4.0 International License, which permits use, sharing, adaptation, distribution and reproduction in any medium or format, as long as you give appropriate credit to the original author(s) and the source, provide a link to the Creative Commons license, and indicate if changes were made.
In cases where reviewers are anonymous, credit should be given to 'Anonymous Referee' and the source.
The images or other third party material in this Peer Review File are included in the article's Creative Commons license, unless indicated otherwise in a credit line to the material. If material is not included in the article's Creative Commons license and your intended use is not permitted by statutory regulation or exceeds the permitted use, you will need to obtain permission directly from the copyright holder.
To view a copy of this license, visit https://creativecommons.org/licenses/by/4.0/

**Reviewers' comments:**

Reviewer #1 (Remarks to the Author):

This manuscript reports a robust, population-based cross-sectional study that characterizes the ocular surface microbiome (OSM) in 1,006 children and adolescents in Hong Kong using 16S rRNA sequencing. The authors successfully identify five distinct microbial clusters ("ocular types") through PAM-based clustering and investigate their associations with demographic factors and ocular parameters, along with functional pathway predictions. However, the proposed ocular "types" would benefit from longitudinal validation and additional functional correlations, such as immune profiling or clinical outcome data, to strengthen their relevance in clinical practice.

**Response**: Thank you for your thoughtful and constructive feedback. We agree that longitudinal validation and additional functional correlations would significantly strengthen the clinical relevance of the proposed ocular types.

1. Regarding the need for longitudinal validation: We acknowledge that our cross-sectional design is a snapshot that cannot assess the temporal stability of these ocular types. To address this important point, we have expanded the Discussion section to explicitly state this as a key limitation.

**Change in the Manuscript: L309-311** "While age is a well-recognized determinant of gut and skin microbiomes, its role in shaping the pediatric OSM need confirmation in longitudinal cohorts spanning a broader age range."

**L337-339** "First, the cross-sectional design restricts our ability to track changes in ocular types over time, preventing the assessment of age-related effects."

2. Regarding the need for additional functional correlations: We agree that linking microbial community structure to host immune response and hard clinical endpoints is the critical next step. In the **Results** section, we now more prominently highlight the association found between Ocular Type 4 (uncultured Neisseriaceae-dominant) and dry eye symptoms (based on OSDI scores) in our cohort, which aligns with the validation from the external dry eye dataset. This directly links a specific ocular type to a prevalent clinical phenotype. In the **Discussion** section regarding functional prediction, we have added an interpretation of the key differential metabolic pathways identified (e.g., Kdo2-lipid A biosynthesis linked to pro-inflammatory potential, aerobactin biosynthesis linked to iron competition). This provides a hypothesized mechanistic bridge between community taxonomy and potential host interaction.

**Change in the Manuscript: L253-259:** "Analysis of the external dataset from the study on dry eye in an Australian population found an association between the ocular type characterized by uncultured *Neisseriaceae* and dry eye disease (Supplementary Table 5). Consistent with this finding, analysis of the OSDI scores within our own cohort revealed a difference in the proportion of participants with dry eye across the five ocular types (Kruskal-Wallis rank sum test, $P$=0.03). Notably, the lowest prevalence of non-dry eye symptoms was observed in Ocular Type 4

(uncultured *Neisseriaceae*-dominant)."

**L330-336:** "Even so, several distinguishing pathways, including aerobactin biosynthesis; superpathway of Kdo2 lipid A biosynthesis; 5-aminoimidazole ribonucleotide biosynthesis routes; and guanosine ribonucleotide de novo biosynthesis, were differentially enriched across Ocular Types, suggesting community-specific metabolic priorities. Such differences may have implications for host–microbe interactions, including epithelial homeostasis, immune regulation, and response to environmental stressors."

**L341-343** "Shotgun metagenomics, metatranscriptomics with host immune profiling would enable provide higher-resolution taxonomic (including strain-level) and functional insights."

Thank you once again for your insightful comment.

Reviewer #2 (Remarks to the Author):

Please mention the IEC approval no. Please clearly explain the inclusion criteria. Please mention the main parameters considered for the comprehensive ophthalmic and physical examinations. Were the kids selected randomly or consecutive enrolment? The age-group considered, and the subgroups based on age?

**Response**: Thank you for these important methodological clarifications. We have revised the manuscript to address each of your points as detailed below:

**1. IEC Approval Number:**

We have updated the **Methods** section (Study Population and Design subsection) to explicitly include the approval number. The sentence now reads: "The study protocol was approved by the Ethics Committee Board of the Chinese University of Hong Kong (Reference No. 2015.033)."

**Change in the Manuscript L118 and Declarations**

**2. Clear Explanation of Inclusion Criteria:**

The **Methods** section has been amended to specify the inclusion criteria. The relevant text now states: " All participants of HKCES from May 2022 to December 2023 aged 3-18 years were invited to join this study. Exclusion criteria consisted of a history of ocular trauma, congenital ocular malformations, inability to complete the examination protocol, and the use of topical eye drops or contact lenses within one week prior to sampling."

**Change in the Manuscript: L101-113**

**3. Main Parameters for Comprehensive Examinations:**

We have expanded the description in the **Methods** to list the key parameters unambiguously. The revised text is: " Enrolled children underwent comprehensive ophthalmic examinations physical examinations. Ophthalmic examinations included cycloplegic autorefraction, axial length, central corneal thickness measurement and other ocular biometry. Physical examination included the measurement of height and weight for the calculation of body mass index (BMI)"

**Change in the Manuscript: L104-109**

**4. Sampling Method (Random vs. Consecutive):**

We have clarified the sampling strategy in the **Methods**. The text now explicitly

states: " This cross-sectional analysis enrolled participants from the Hong Kong Children Eye Study (HKCES), an ongoing, multi-ethnic, population-based cohort investigating ocular health in schoolchildren. Participant recruitment employed a **stratified, clustered random sampling** strategy across seven geographical districts in Hong Kong."

**Change in the Manuscript: L101-104**

5. Age-Group and Subgroups:

We have clarified the age range in the **Methods**. The **Results** section states the cohort includes participants "aged 3 to 18 years." Age was analyzed as a continuous variable in correlation analyses with ocular types. And for the subgroups analysis was based on randomly sampling from the whole dataset.

**Change in the Manuscript: L110:** "All participants of HKCES from May 2022 to December 2023 aged 3-18 years were invited to join this study."

**L157-159:** "To verify the reliability of our clustering outcome (ocular type), we divided our dataset randomly into two subsets, allocating 60% and 40% to each, and assessed the consistency between the results of these two subsets."

How was it selected if one or both eyes of patients will be sampled? Was a smaller size paediatric swab used for the collection?

**Response**: Thank you for these important questions regarding our sampling methodology.

1. Selection of One or Both Eyes for Sampling:

   Our sampling strategy was refined in response to preliminary data. In the initial phase of the study, conjunctival samples were collected from both eyes of participants. Comparative analysis of these paired samples (n=240) revealed no significant difference in microbial composition between the right and left eyes of the same individual. Given this finding of high inter-ocular similarity, we adopted a more cost-effective way to increase participant enrolment sampling only the right eye for sequencing (Left-eye samples were still collected but were used only when the corresponding right-eye sample did not meet quality-control requirements). This rationale and the supporting analytical results are now clearly stated in the **Results** section.

2. Specification of the Swab Used:

   A standard, commercially available sterile flocked swab was used for all collections: the **6-inch HydraFlock Sterile Straight Flock Swab** (Catalog No. 25-3000-H E30). This swab type is appropriate for pediatric use and is designed for effective sample collection. There is a breakpoint 30mm from tip, allowing the swab head to be easily aseptically detached into the collection tube to minimize contamination.

**Change in the Manuscript: L121-123:** "a sterile individually packaged Puritan flocked swab (Catalog No. 25-3000-H E30) was used to swab the inferior conjunctival fornix by gently rolling from the nasal to temporal sides and then back for at least 3 rounds."

Was the nutritional status of the kids and hygiene (general and ocular) taken into consideration during analysis as this might also alter the surface microbiome?

**Response**: Thank you for this excellent and highly relevant point regarding potential confounders of the ocular surface microbiome.

We acknowledge that nutritional status and hygiene practices are important factors that could influence microbial composition. In our study, we collected physical measurements (height and weight) for all participants. These data enabled the calculation of body mass index (BMI), a commonly used proxy for nutritional status in microbiome research, including studies of the gut microbiome. This variable was included in our correlation analyses with the ocular microbiome types; however, no significant associations were found between the ocular types and BMI in our cohort.

Regarding hygiene, while we did not collect detailed questionnaire data on general or ocular-specific hygiene habits, our exclusion criteria were designed to minimize the acute impact of ocular hygiene interventions. We excluded participants who had used any topical eye drops or contact lenses within the week prior to sampling. This was intended to control for recent direct chemical or physical alteration of the ocular surface environment.

We agree that more granular data on dietary patterns, hygiene frequency, and use of cleansing products would provide a more complete picture. The absence of this data is a recognized limitation of the current cross-sectional, population-based design. We have added this point in the revised **Discussion** section, stating that future, more detailed studies should incorporate standardized assessments of nutrition and hygiene to better understand their modulating effects on the ocular surface microbiome structure we have described.

**Change in the Manuscript: L312-314:** "Despite links between nutrition and microbial community structure in the gut, BMI did not differ across Ocular Types in our cohort (*P*=0.22, ANOVA test, Supplementary Table 7)."

**L113-115:** "Exclusion criteria consisted of a history of ocular trauma, congenital ocular malformations, inability to complete the examination protocol, and the use of topical eye drops or contact lenses within one week prior to sampling."

**L344-347:** "missing covariates including general or ocular hygiene practices and environmental exposures limit the granularity of association analyses, which will require more comprehensive data collection in future studies to investigate their impact on ocular microbial variation."

Uncultured Neisseriaceae was known to be associated with dry eye. Did these kids in this cohort with this organism have any dry eye?

**Response**: Thank you for this important question regarding the clinical correlation of the uncultured *Neisseriaceae*-dominant ocular type. We have added this analysis now.

Yes, within our cohort, participants with ocular type characterized by

uncultured *Neisseriaceae* (Ocular Type 4) showed a significant association with dry eye symptoms. This was assessed using the Ocular Surface Disease Index (OSDI) questionnaire.

**Change in the Manuscript: L253-259** "Analysis of the external dataset from the study on dry eye in an Australian population found an association between the ocular type characterized by uncultured *Neisseriaceae* and dry eye disease (Supplementary Table 5). Consistent with this finding, analysis of the OSDI scores within our own cohort revealed a difference in the proportion of participants with dry eye across the five ocular types (Kruskal-Wallis rank sum test, $P$=0.03). Notably, the lowest prevalence of non-dry eye symptoms was observed in Ocular Type 4 (uncultured *Neisseriaceae*-dominant)."

L222-230: For the functional prediction of ocular types, please explain the significance of finding these associated metabolic pathways.

**Response**: Thank you for this insightful question. The identification of differentially abundant metabolic pathways among ocular types provides important functional context that moves beyond taxonomic description. It suggests how these distinct microbial communities might interact with and influence the ocular surface environment. We have now added explanation in Discussion session.

**Change in the Manuscript: L326-336** "Predicted functional profiling showed that, despite compositional differences, Ocular Types shared a large overlap in pathway space on PCoA, points towards a potential "functional core". Defining such core functions may guide the design of microbiome-targeted interventions to restore "healthy microbiome" in dysbiotic states. Even so, several distinguishing pathways, including aerobactin biosynthesis; superpathway of Kdo2 lipid A biosynthesis; 5-aminoimidazole ribonucleotide biosynthesis routes; and guanosine ribonucleotide de novo biosynthesis, were differentially enriched across Ocular Types, suggesting community-specific metabolic priorities. Such differences may have implications for host–microbe interactions, including epithelial homeostasis, immune regulation, and response to environmental stressors."

Discussion

The authors clearly explained here that the purpose of this study was to classify the 5 types of OSM in children in Hongkong with different ethnicities, so that it would serve as a reference for future studies. But it would be good if they did some more correlation of the data to show how different OSM was related to different ethnicities' ocular surface diseases in that age group from any previous publications and how it was related to nutritional status of patients etc.

**Response**: Thank you for the insightful comment. We appreciate the suggestion to provide deeper correlations between our identified Ocular Types, ethnicity, and ocular surface diseases.

We agree that establishing direct correlations between specific Ocular Type and the prevalence of ocular surface diseases within each ethnic subgroup is a

compelling research direction. Our study's primary aim was to establish a foundational taxonomic framework, the five Ocular Type within a multi-ethnic pediatric cohort. While we analyzed key demographic associations, a detailed, disease-specific epidemiological analysis across ethnicities was beyond the scope of this initial characterization. Future longitudinal studies incorporating detailed clinical phenotyping are essential to investigate whether certain Ocular Type confer increased risk for particular diseases within specific ethnic groups.

We incorporated Body Mass Index (BMI) as a proxy for nutritional status in our correlation analyses. In our cohort, no significant association was found between any Ocular Microbiome Type and BMI. This suggests that within the generally healthy population studied, broad nutritional status may be a less dominant driver of ocular surface microbial community structure than factors like ethnicity or age. However, we recognize that BMI is a crude measure, and more granular data on diet, micronutrient levels, or metabolic markers in future studies could reveal more subtle relationships.

We appreciate the suggestion to cross-validate our findings against prior datasets. We attempted this validation step; however, a formal pooled analysis across external datasets was not feasible for three key reasons: 1. Publicly available 16S rRNA datasets with multiple ethnicities are scarce; 2. Many existing datasets do not report or capture detailed ethnicity data, which precludes the subgroup analysis requested; 3. Differences in sampling methods (conjunctival swab vs. tear collection), DNA extraction kits, sequencing platforms, bioinformatic pipelines and geography between studies introduce significant technical variation. Simply merging such data would confound true biological signals with methodological noise. Accordingly, rather than aggregating incompatible datasets, we expanded the Discussion to review the limited multi-ethnic literature, noted these constraints as a study limitation, and highlighted the need for well-designed, multi-ethnic pediatric cohorts with detailed clinical phenotyping to rigorously evaluate ethnicity-related differences.

**Change in the Manuscript: L312-325** "Despite links between nutrition and microbial community structure in the gut, BMI did not differ across Ocular Types in our cohort (P=0.22, ANOVA test, Supplementary Table 7). This is biologically plausible given the limited direct nutritional exposure of the conjunctival surface, although systemic factors cannot be excluded. We also find ethnic associations that all Ocular Type 5 samples occurred in Chinese children, while Nepalese children were enriched for Ocular Type 2, pointing to possible contributions from genetic background, environment, and lifestyle. Previous research on ethnic variation in the ocular microbial composition is limited, with only one culture-based study reporting differences between two Chinese ethnic groups. Directly comparing microbial compositions across distinct geographical regions is challenging due to geography itself being a significant confounder. Therefore, a well-designed, multi-ethnic study is required to validate these ethnicity-related findings, which may provide critical insights into disease risk and differential treatment responses."

Overall, the work, though innovative, needs reanalysis and further correlation

studies and literature review to make the findings more robust and impactful.

**Response**: We sincerely thank the reviewer for the insightful comment and suggestion, which have helped improve the manuscript. In this revision, we have incorporated additional analyses and expanded the correlation assessments. We hope these revisions modestly enhance the clarity of the work.

Reviewer #3 (Remarks to the Author):

This study aimed to define ocular types in a cohort of children aged between 3 and 18 years. It profits from a large sample size but suffers from a small age range to do adequate statement about age-related findings. For details, see the following concerns.

**Response**: We thank the reviewer for the thoughtful comments which help us improve the manuscript. We have revised the manuscript accordingly.

Major concerns:

1) The age range is too small to make a statement about age-related ocular types. To assess this, the cohort should include participants of a larger age range.

**Response**: Thank you for highlighting this important concern. Our study was designed to characterize the pediatric ocular surface microbiome, a developmental stage that is biologically distinct from adulthood. For this reason, our recruitment criteria focused on children and adolescents aged 3–18 years.

Within this pediatric cohort, we observed that Ocular Type 5 (Escherichia Shigella dominant) appeared only in Chinese children and showed a positive association with age. To explore whether this pattern might extend beyond childhood, we additionally examined an independent Hong Kong adult cohort (n = 130; ages 28–61 years). In adults, Ocular Type 5 was not detected; instead, all samples mapped to Ocular Types 1–4, and age showed a positive association with Ocular Type 2 (Corynebacterium dominant) (Supplementary Table 6).

Taken together, these findings suggest that age may have association with ocular community states, but the evidence remains preliminary and requires further validation. While age is a well-recognized determinant of gut and skin microbiomes, its role in shaping the pediatric ocular surface microbiome remains insufficiently understood. We agree that longitudinal studies spanning a broader age range will be essential for confirming and refining these observations.

**Change in the Manuscript: L302-311** “We observed cluster-specific demographic and ocular associations. Notably, Ocular Type 5 (*Escherichia-Shigella* dominant) occurred only among Chinese children and showed a positive association with age within this pediatric cohort. In an independent Hong Kong adult cohort (n=130, ages 28–61 years), Ocluar Type 5 was not detected; all samples mapped to Ocular Types 1–4, and age was positively associated with Ocular Type 2

(*Corynebacterium*-dominant) (Supplementary Table 6). Taken together, these findings suggest that age might relate to ocular community state. While age is a well-recognized determinant of gut and skin microbiomes, its role in shaping the pediatric OSM need confirmation in longitudinal cohorts spanning a broader age range."

2) Line 176: The absence of significant differences between the right and left eye does not necessarily implicate consistent and robust data. Are the data between the left and right eye more similar that the data of eyes from different participants?

**Response:** The reviewer is absolutely correct. We apologize for the incomplete reporting. We have added this analysis now. The beta diversity (Bray-Curtis dissimilarity) between paired eyes from the same individual was lower than that between randomly selected eyes from different individuals (Mann-Whitney U test, $p < 0.001$). This confirms high interpersonal variation and intrapersonal consistency. We now added this statistical comparison to the **Results** section and a reference to **Supplementary Figure 2D**. Thank you very much for this important suggestion.

**Change in the Manuscript: L201-206** "Paired samples from the right and left eyes of the same participant (n=240) showed no significant difference in microbial composition. (**Supplementary Figure 2**). Furthermore, the beta diversity (Bray-Curtis dissimilarity) between samples from the same individual was significantly lower than that between samples from different individuals (Mann-Whitney U test, $P$<0.001), indicating consistent personal microbial profiles and supporting the robustness of data (**Supplementary Figure 2D**)."

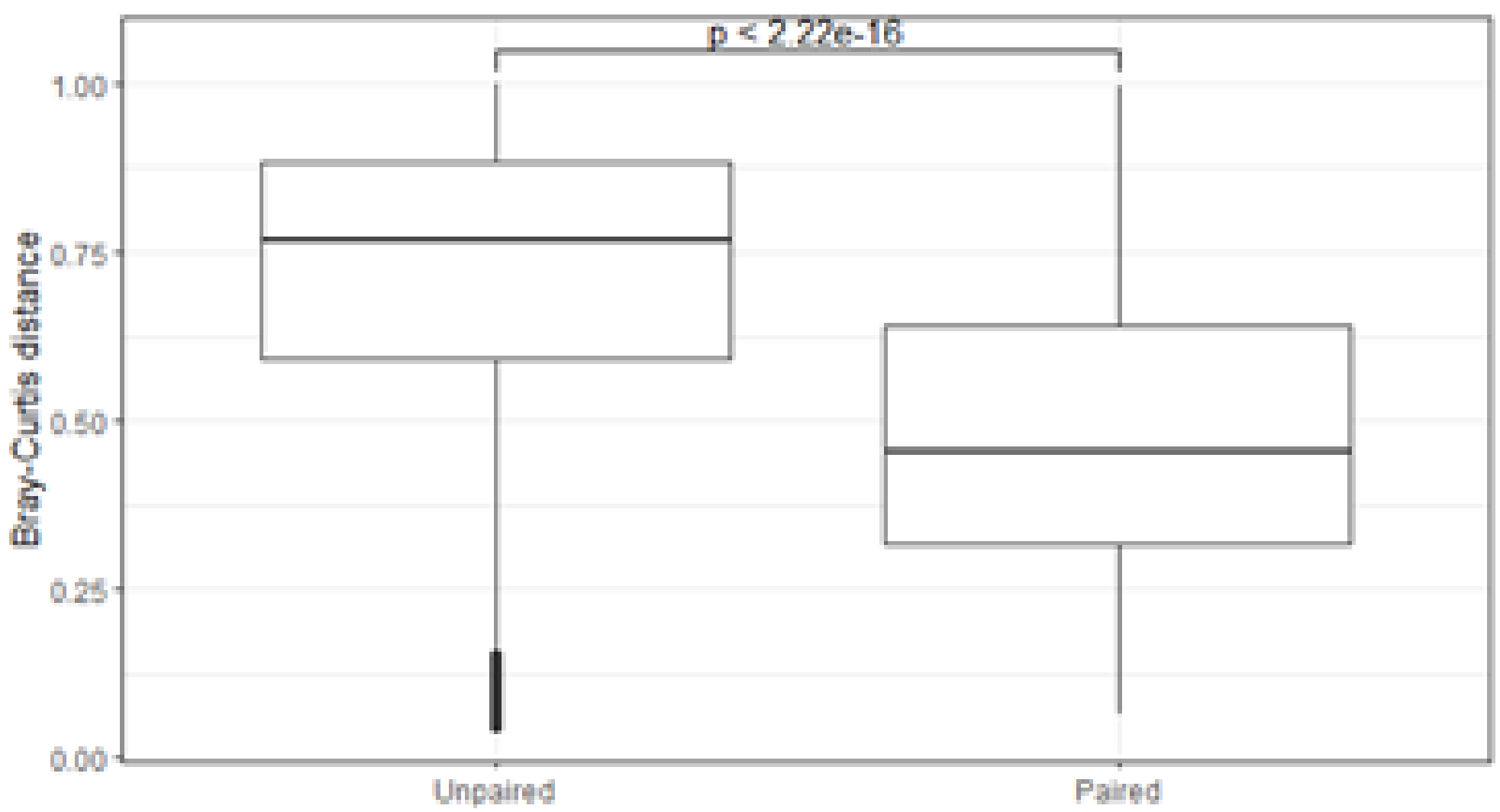


Supplementary Figure 2D

Minor concerns:

1) line 69: The term «microbiome» refers to the microbes and their genetic entities in a defined niche. Please adapt the term accordingly.

Response: Thank you very much. We have revised the opening definition to: “Ocular surface microbiome (OSM) refers to the community of microorganisms and their genetic materials on the surface of the human eye. “

**Change in the Manuscript: L71-72** “Ocular surface microbiome (OSM) refers to the community of microorganisms and their genetic materials on the surface of the human eye.”

2) Missing information:

— inclusion criteria: Was intake of antibiotics in the last few months not an exclusion criteria? If not, was intake of antibiotics recorded during the recruitment process?

Response: Thank you very much for raising this important point. The use of topical eye drops, including antibiotics, within one week prior to sampling was part of our exclusion criteria. In addition, all systemic and topical medication use within the preceding six months was recorded through a questionnaire during recruitment.

**Change in the Manuscript: L113-115** “Exclusion criteria consisted of a history of ocular trauma, congenital ocular malformations, inability to complete the examination protocol, and the use of topical eye drops or contact lenses within one week prior to sampling.”

— How many PCR cycles have been performed (line 120)?

Response: We appreciate the reviewer’ s question. We performed 25 PCR cycles, following the Illumina *16S Metagenomic Sequencing Library Preparation Guide* (Part #15044223 Rev. B). This information has now been added to the Methods section for clarity.

**Change in the Manuscript: L135-140** “PCR amplification of V3-V4 region of bacterial 16S rRNA gene was performed with 341F forward primer (CCTACGGGNGGCWGCAG) and 805R reverse primer (GACTACHVGGGTATCTAATCC) following the 16s Illumina Demonstrated Library Prep Guide (# 15044223 Rev. B). Twenty-five amplification cycles were performed.”

— Which method was used in Decontam for removal of potential contaminants (line 131)?

Response: We thank the reviewer for this question. We used the prevalence-based method in the *decontam* package, applying a threshold of 0.5 to identify and remove potential contaminants by comparing experimental samples with environmental controls. This threshold is relatively stringent, which we considered appropriate given the low-biomass nature of ocular surface samples and our aim to minimize the influence of contaminating taxa. This information

has now been explicitly clarified in the revised Methods section.

**Change in the Manuscript: L147-149** "alongside the *Decontam* package for contamination removal with prevalence-based contaminant identification (threshold=0.5)."

— line 150: why did you use LEfSe for taxonomic analysis and Maaslin2 for pathway analysis? Which parameter did you use for both of them?

Response: **LEfSe** was chosen for identifying **taxonomic biomarker** across our five predefined Ocular Types (clusters). It is well-suited for identifying features that characterize multiple, discrete groups. We used default parameters with an LDA score threshold of >4.0 and a Kruskal-Wallis test significance of $p < 0.01$. **Maaslin2** was used for **functional pathway analysis** to test associations between pathway abundance and ocular type as a fixed effect, allowing for normalization and transformation of the compositional data. We used default parameters (normalization: "TSS", transformation: "log", analysis method: "LM").

**Change in the Manuscript: L219-223** "Discriminant markers among the ocular types were identified via LEfSe (Supplementary Fig 5, LDA score >4, $P$<0.01 for Kruskal-Wallis Test), revealing dominant genera including Staphylococcus (Ocular Type 1), Corynebacterium (Ocular Type 2), Streptococcus (Ocular Type 3), uncultured Neisseriaceae (Ocular Type 4), and Escherichia-Shigella (Ocular Type 5; **Figure 2F**)."

**L168-170** "The metabolic pathway abundance was predicted by PICRUSt2[31] and analyzed by *Maaslin2* package with default parameter (normalization: "TSS"; transformation: "log"; analysis method: "LM")"

— Line: 207: How were dry eyes assessed?

Response: Our internal dataset assessed dry eye symptoms were assessed using the validated Ocular Surface Disease Index (OSDI) questionnaire. The cutoff scores are 0-12, 13-22, 23-32, 33-100. The external dataset (Ozkan et al. Experimental Eye Research 2023) used diagnostic criteria according to the Tear Film and Ocular Surface Society (TFOS) Dry Eye Workshop (DEWS) II Diagnostic Methodology Report.

**Change in the Manuscript: L109-111** "Dry-eye symptoms were assessed using the validated Ocular Surface Disease Index (OSDI) questionnaire.24 Standard thresholds were applied (0–12 normal, 13–22 mild, 23–32 moderate, 33–100 severe)."

3) line 161: the cohort is not balanced between the different ethnicities.

Response: We agree with the reviewer that the cohort is not balanced across ethnicities, with Chinese children comprising the majority. This reflects the demographic reality of Hong Kong's school-age population. Our stratified, clustered random sampling was designed to capture a population-representative sample, therefore failed to achieve equal group sizes. The primary aim was to establish ocular microbiome types in a general pediatric population. Although we observed some ethnicity-related patterns (Type 5 exclusive to Chinese, Type 2 correlated with Nepalese), we acknowledge that these findings require further

validation in larger, multi-ethnic pediatric cohorts.

**Change in the Manuscript: L322-325** "Therefore, a well-designed, multi-ethnic study is required to validate these ethnicity-related findings, which may provide critical insights into disease risk and differential treatment responses."

4) line 173: is the term "core taxa" according to your definition of a detection level of 1% and a prevalence of 50%? If yes, is this definition in accordance with previously published ocular surface microbiome data?

Response: Yes, our definition of "core taxa" is based on a combined threshold of >1% relative abundance in at least one sample and >50% prevalence across all samples. This approach is consistent with recently published ocular surface microbiome studies. Morandi et al. (*Invest Ophthalmol Vis Sci*, 2025) removed species with <1% relative abundance as likely contaminants and then applied a 50% prevalence cutoff to define the core ocular microbiome. Our previous work (Ling et al., *Eye (Lond)*, 2025) also used the same criteria. These supporting publications have now been cited in the revised manuscript.

**Change in the Manuscript: L148:** "Core taxa were defined by a detection level of 1% and a prevalence of 50%."

**Reviewers' comments:**
Reviewer #1 (Remarks to the Author):
The manuscript has been thoroughly revised to address all reviewers' comments and is now ready for publication.

**Response**: Thank you very much for your guidance that improved the manuscript.

Reviewer #3 (Remarks to the Author):

Thank you for providing the revised manuscript that improved a lot, mainly by adding the older age cohort. However, I still have some concern that have to be considered.

**Response**: We greatly appreciate your thoughtful and constructive concerns, which have helped us further strengthen the work. Please find our point-by-point responses below.

1) It remains unclear why different statistical tools were applied for the taxonomic and functional analyses, specifically the use of LEfSe for taxonomic composition and MaAsLin2 for pathway analysis. This choice raises concerns regarding methodological consistency and comparability of results within the same study.
MaAsLin2 is specifically designed to detect associations between microbial features and metadata variables within a multivariable modeling framework. Importantly, it allows adjustment for potential confounders and supports the inclusion of random effects, thereby accounting for complex study designs and reducing the risk of biased associations. In contrast, LEfSe is primarily a univariate biomarker discovery tool and does not provide a framework for adjusting for confounding variables.
Given these fundamental differences in statistical approach, the rationale for applying LEfSe to taxonomic data while using MaAsLin2 for pathway analysis requires clearer justification. If confounding factors were considered relevant for the pathway analysis, they should arguably also have been incorporated into the taxonomic analysis to ensure methodological coherence and internal consistency of the findings.
Furthermore, the manuscript should provide a detailed description of the MaAsLin2

model specifications. Please explicitly state:
Which variables were included as fixed effects?
Whether random effects were incorporated, and if so, which ones?
Providing these details is essential for transparency, reproducibility, and proper interpretation of the reported associations.

**Response:** Thank you for raising this important methodological point. We agree that consistency in statistical approaches is crucial for internal coherence. After careful consideration of your comment, we have **revised our analyses to use LEfSe for both taxonomic composition and pathway analysis**. This ensures that the same statistical framework is applied across all microbial features, making the results directly comparable.

**Change in the Manuscript: L171-174** "Differentially abundant taxa across the defined ocular types were identified using Linear discriminant analysis (LDA) Effect Size (LEfSe). The metabolic pathway abundance was predicted by PICRUSt2[35] and compared by LEfSe."

Supplementary Figure 26 has been updated and the pathways with LDA score >3 (*P*<0.01 for Kruskal-Wallis Test) were shown:

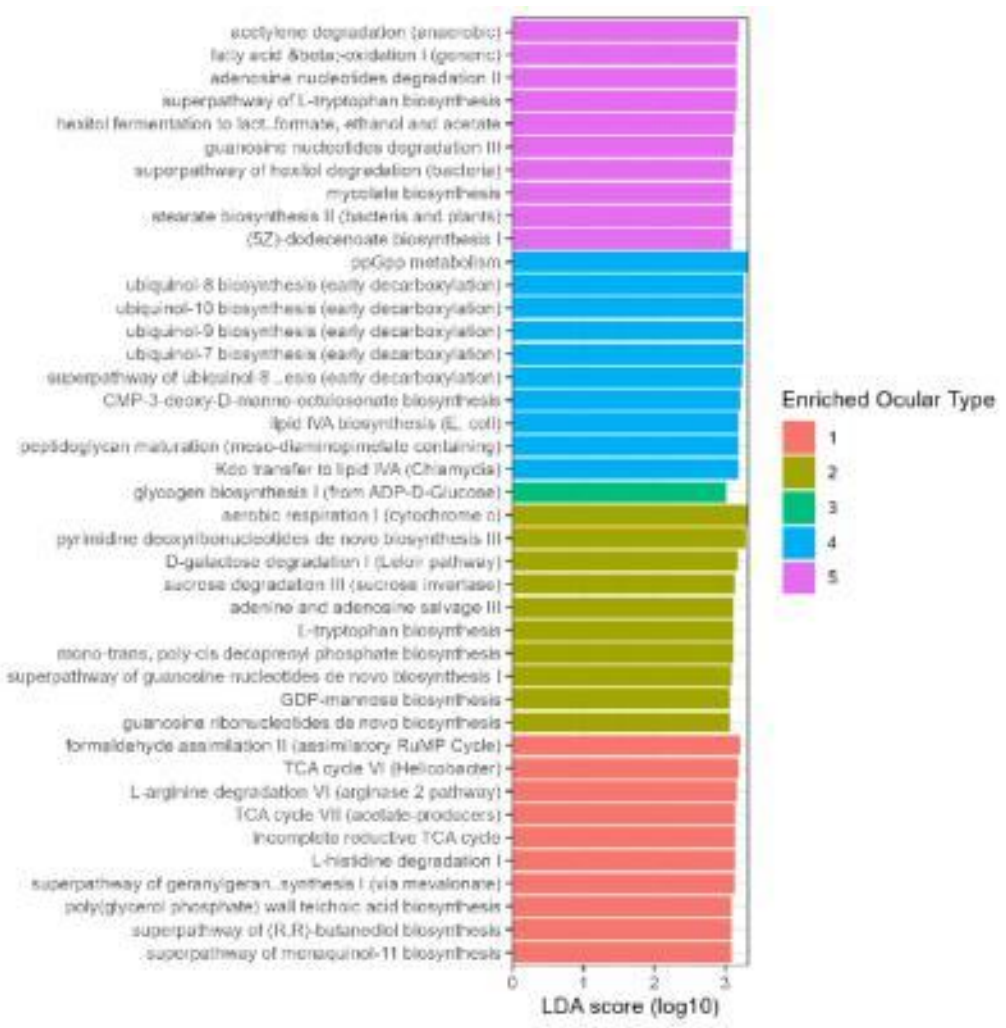


2) The use of the OSDI questionnaire in the present study requires further justification. The OSDI was originally developed and validated for adult populations and has not been specifically validated for use in children. Consequently, its applicability and validity in a pediatric cohort are questionable. Several items included in the questionnaire may not be age-appropriate and could compromise the reliability of the collected data. For example, questions referring to "difficulty driving at night" are clearly not applicable to children. In addition, younger children may have limited ability to accurately self-assess and quantify subjective ocular symptoms, further affecting the validity of the responses. Given these limitations, the authors should clarify whether a modified or adapted version of the OSDI was used and how age-appropriateness was ensured. Alternatively, the use of a pediatric-

validated instrument would be more methodologically sound.

**Response:** We appreciate this important point. In our cohort, we used an adapted OSDI protocol consistent with prior pediatric use (Chidi-Egboka et al. Ophthalmic Physiologic Optic 2020). Specifically, the “driving at night” item (Q7) was omitted as not age-appropriate, and the final score was calculated using the standard OSDI formula that adjusts for the number of answered questions. For younger participants, trained staff provided standardized assistance/parental support to ensure comprehension without leading responses. This approach is supported by pediatric work indicating that OSDI-based questionnaires (with inapplicable items removed) can be administered reliably in children (n = 62; 25M:37F; aged 6-15 years), but sometimes requiring additional assistance for younger ages. We have added these details and the supporting citation in the Methods.

**Change in the Manuscript: L110-115** “Dry-eye symptoms were assessed using Ocular Surface Disease Index (OSDI) questionnaire.[24] One question (Q7, Driving at night) (Q7, "Driving at night") was deemed irrelevant to the pediatric population and was omitted.[25] The final score was calculated using the standard formula (sum of scores × 25 / number of questions answered) to account for the omitted item. Supplemental assistance was provided to children who required support in completing the questionnaire.”

3) If the term “core taxa” is based on already published literature, please add the corresponding literature (Morandi et al.). It may be better to use “core microbiota” or “core microbiome” instead of “core taxa”.

**Response:** Thank you for this helpful suggestion. We have replaced all instances of “core taxa” with “core microbiota” throughout the manuscript and added the citation to Morandi et al. to support the definition and usage.

**Change in the Manuscript: L43-46** “Here we show that in 1,246 samples from 1,006 individuals aged 3 to 18 years, the predominant bacterial phyla are Proteobacteria (34.1%), Firmicutes (37.4%), and Actinobacteria (25.2%), with *Staphylococcus*, *Corynebacterium*, and *Streptococcus* as core microbiota.”

**L155-156** “Core microbiota was defined by a detection level of 1% and a prevalence of 50%31,32.”
