# Supplementary material for: Profiling of ocular surface microbiome and its ocular types in children and adolescents
Source: Commun Med (Lond). 2026 Jun 8;6:487. doi: 10.1038/s43856-026-01667-7 (PMC13582937; doi:10.1038/s43856-026-01667-7)

Supplementary Table 1. Relative abundance of 5 predominated genera in clusters of two subsets.

Ocular type	Genus	Subset 1 (60%)		Subset 2 (40%)	
		Mean	SE	Mean	SE
Ocular type Staphylococcus	Corynebacterium	0.059179	0.00558	0.078478	0.007611
	Escherichia-Shigella	0.009458	0.003851	0.016511	0.004615
	**Staphylococcus**	**0.63507**	0.012492	**0.5685**	0.015961
	Streptococcus	0.047866	0.004944	0.035329	0.003835
	uncultured_Neisseriaceae	0.017078	0.00352	0.013765	0.00402
Ocular type Corynebacterium	**Corynebacterium**	**0.59325**	0.017659	**0.55761**	0.019527
	Escherichia-Shigella	0.012281	0.005078	0.007722	0.003223
	Staphylococcus	0.131467	0.013443	0.115183	0.012665
	Streptococcus	0.027683	0.004006	0.041014	0.005742
	uncultured_Neisseriaceae	0.020893	0.005748	0.036941	0.009505
Ocular type Streptococcus	Corynebacterium	0.075519	0.004497	0.055188	0.005004
	Escherichia-Shigella	0.03039	0.004093	0.012975	0.002716
	Staphylococcus	0.138226	0.007403	0.095683	0.007158
	**Streptococcus**	**0.11529**	0.00743	**0.14087**	0.012112
	uncultured_Neisseriaceae	0.013591	0.002178	0.015387	0.00359
Ocular type Uncultured Neisseriaceae	Corynebacterium	0.090515	0.01127	0.049157	0.010189
	Escherichia-Shigella	0.008161	0.002922	0.012313	0.007708
	Staphylococcus	0.106059	0.01245	0.133433	0.019753
	Streptococcus	0.033185	0.005578	0.020017	0.00498
	**uncultured_Neisseriaceae**	**0.57032**	0.02144	**0.60101**	0.029291
Ocular type Escherichia-Shigella	Corynebacterium	0.029776	0.006189	0.03138	0.009214
	**Escherichia-Shigella**	**0.68788**	0.034648	**0.72444**	0.033929
	Staphylococcus	0.024382	0.005696	0.068245	0.01495
	Streptococcus	0.017909	0.007994	0.00721	0.002507
	uncultured_Neisseriaceae	0.016444	0.009138	0.011366	0.004534

Bold font indicates the representative genus of the Ocular type and its proportion.

Supplementary Table 2. Demographic data of ocular types in two subsets.

Ocular type	Percentages in subset		Age (mean ± SD)		Male %	
	Subset 1	Subset 2	Subset 1	Subset 2	Subset 1	Subset 2
Ocular type Staphylococcus	23.0%	26.1%	10.66 ± 2.20	10.92 ± 2.61	62.0%	51.9%
Ocular type Corynebacterium	14.9%	17.8%	9.64±2.65	9.69 ± 2.55	55.5%	51.7%
Ocular type Streptococcus	41.8%	37.3%	10.36 ± 2.80	9.95 ± 2.61	53.9%	47.3%
Ocular type Uncultured Neisseriaceae	13.0%	10.4%	10.49 ± 2.43	9.90 ± 2.61	50.5%	61.5%
Ocular type Escherichia-Shigella	7.36%	8.41%	10.24 ± 2.25	11.19 ± 2.85	51.9%	52.4%

Supplementary Table 3. Relative abundance of 3 predominated genera in ocular types of external dataset 1 (Yau's dataset on ARC).

Ocular type	Genus	Mean of relative abundance	SE of relative abundance
1	**Staphylococcus**	**0.1715**	0.031868
1	Streptococcus	0.07361	0.021343
1	unknown_Family_Neisseriaceae	0.028098	0.01483
2	Staphylococcus	0.03661	0.006056
2	**Streptococcus**	**0.234521**	0.058234
2	unknown_Family_Neisseriaceae	0.004094	0.001499
3	Staphylococcus	0.002763	0.001717
3	Streptococcus	0.093056	0.008244
3	**unknown_Family_Neisseriaceae**	**0.73199**	0.112303

Bold font indicates the representative genus of the Ocular type and its proportion.

Supplementary Table 4. Relative abundance of 3 predominated genera in ocular types of external dataset 2 (Ozkan's dataset on dry eye).

Ocular type	Genus	Mean of relative abundance	SE of relative abundance
1	**Corynebacterium**	**0.531762**	0.021286
1	QFNR01	0.031206	0.007709
1	Staphylococcus	0.122425	0.011754
2	Corynebacterium	0.056749	0.005411
2	QFNR01	0.011034	0.003188
2	**Staphylococcus**	**0.190082**	0.019627
3	Corynebacterium	0.073005	0.01539
3	**QFNR01**	**0.663284**	0.034829
3	Staphylococcus	0.045115	0.008127

Bold font indicates the representative genus of the Ocular type and its proportion.

Supplementary Table 5. Correlation between ocular types and the diagnosis in external dataset 2.

Site	Ocular type	MGD_LD	MGD	control
Conjunctiva	1 (Corynebacterium)	-0.5533	0.8148	-1.1285 **
	2 (Staphylococcus)	-0.1361	-1.2347 **	1.0116 **
	3 (QFNR01)	1.946	1.912	-3.784 ***
Lid margin	1 (Corynebacterium)	-0.7376	3.242e-16	0.04445
	2 (Staphylococcus)	0.2877	-0.6061	-0.4055
	3 (QFNR01)	0.9163	1.0745	-2.3273 ***

The logistic regression analysis was conducted with the diseased groups of the samples. The dependent variable indicates whether the child belongs to the ocular type or not. The significance levels are denoted by asterisks (*), where * indicates $p < 0.05$, ** indicates $p < 0.01$, and *** indicates $p < 0.001$.

Supplementary Table 6. Association between age and Ocular Type distribution in a separated Hong Kong adult cohort.

Ocular Type	1	2	3	4
Representative genus	Staphylococcus	Corynebacterium	Streptococcus	uncultured Neisseriaceae
N	35	38	27	30
Age	42.9±6.1	45.9±5.8	41.9±5.2	42.7±6.1
P value*	0.71	0.005	0.10	0.32

* Logistic regression analysis was performed to examine the association between Age and the probability of belonging to the specified Ocular Type.

Supplementary Table 7. Analysis of variance (ANOVA) results comparing the BMI of different Ocular Types.

Source	Degrees of Freedom (Df)	Sum of Squares (Sum Sq)	Mean Square (Mean Sq)	F value	Pr(>F)
Ocular Type	4	71.00	17.85	1.439	0.219
Residuals	1166	14465.00	12.40		

Supplementary Table 8. Correlation between ocular types with demographic data, ocular biometrics and OSM diversity. AL = Axial Length, ACD = Anterior Chamber Depth, K1 = Keratometry Reading 1, K2 = Keratometry Reading 2, CCT = Central Corneal Thickness, IOP = Intraocular Pressure, with significance denoted by (*p < 0.05, **p < 0.01, ***p < 0.001)

Var1	Var2	r	p_value	signif
Oclaur Type 1	Age	-0.04027	0.302423	
Oclaur Type 2	Age	-0.00913	0.747712	
Oclaur Type 3	Age	0.012598	0.747712	
Oclaur Type 4	Age	-0.03793	0.302423	
Oclaur Type 5	Age	0.106565	0.000836	***
Oclaur Type 1	Male	0.021605	0.711073	
Oclaur Type 2	Male	0.016221	0.711073	
Oclaur Type 3	Male	0.004539	0.873337	
Oclaur Type 4	Male	-0.02644	0.711073	
Oclaur Type 5	Male	-0.03082	0.711073	
Oclaur Type 1	Chinese	0.001008	0.97164	
Oclaur Type 2	Chinese	-0.0758	0.018585	*
Oclaur Type 3	Chinese	-0.02455	0.644136	
Oclaur Type 4	Chinese	-0.0016	0.97164	
Oclaur Type 5	Chinese	0.146399	1.05E-06	***
Oclaur Type 1	Pakistani	0.021305	0.754056	
Oclaur Type 2	Pakistani	0.000987	0.972227	
Oclaur Type 3	Pakistani	0.008473	0.956363	
Oclaur Type 4	Pakistani	0.022913	0.754056	
Oclaur Type 5	Pakistani	-0.08341	0.016079	*
Oclaur Type 1	Nepalese	-0.00891	0.954009	
Oclaur Type 2	Nepalese	0.076246	0.017723	*
Oclaur Type 3	Nepalese	0.001635	0.954009	
Oclaur Type 4	Nepalese	-0.00759	0.954009	
Oclaur Type 5	Nepalese	-0.07878	0.017723	*
Oclaur Type 1	Indian	0.002956	0.91697	
Oclaur Type 2	Indian	0.012638	0.819785	
Oclaur Type 3	Indian	0.026014	0.819785	
Oclaur Type 4	Indian	-0.01924	0.819785	
Oclaur Type 5	Indian	-0.04435	0.588307	
Oclaur Type 1	Sphere	0.002921	0.924459	
Oclaur Type 2	Sphere	-0.01608	0.752187	
Oclaur Type 3	Sphere	0.040916	0.459963	
Oclaur Type 4	Sphere	0.025928	0.666595	
Oclaur Type 5	Sphere	-0.09966	0.005916	**
Oclaur Type 1	Cylinder	0.002317	0.940041	
Oclaur Type 2	Cylinder	0.027887	0.815494	
Oclaur Type 3	Cylinder	0.0213	0.815494	
Oclaur Type 4	Cylinder	0.012599	0.853207	

Ocluar Type 5	Cylinder	-0.10219	0.004414	**
Ocluar Type 1	AL	0.01513	0.62549	
Ocluar Type 2	AL	0.028206	0.453522	
Ocluar Type 3	AL	-0.03232	0.453522	
Ocluar Type 4	AL	-0.0617	0.115886	
Ocluar Type 5	AL	0.084939	0.030271	*
Ocluar Type 1	K1	-0.01801	0.806985	
Ocluar Type 2	K1	-0.01004	0.806985	
Ocluar Type 3	K1	-0.01016	0.806985	
Ocluar Type 4	K1	0.041552	0.806985	
Ocluar Type 5	K1	0.007629	0.806985	
Ocluar Type 1	K2	-0.01321	0.705375	
Ocluar Type 2	K2	-0.02904	0.587656	
Ocluar Type 3	K2	-0.01181	0.705375	
Ocluar Type 4	K2	0.032838	0.587656	
Ocluar Type 5	K2	0.041223	0.587656	
Ocluar Type 1	ACD	0.063	0.210648	
Ocluar Type 2	ACD	0.034103	0.339546	
Ocluar Type 3	ACD	-0.04769	0.310377	
Ocluar Type 4	ACD	-0.0202	0.515095	
Ocluar Type 5	ACD	-0.03683	0.339546	
Ocluar Type 1	IOP	-0.00228	0.941937	
Ocluar Type 2	IOP	0.008979	0.941937	
Ocluar Type 3	IOP	0.014582	0.941937	
Ocluar Type 4	IOP	-0.05209	0.479249	
Ocluar Type 5	IOP	0.036405	0.611703	
Ocluar Type 1	CCT	0.048417	0.175454	
Ocluar Type 2	CCT	-0.02434	0.43784	
Ocluar Type 3	CCT	0.046244	0.175454	
Ocluar Type 4	CCT	-0.04728	0.175454	
Ocluar Type 5	CCT	-0.07717	0.068945	

Supplementary Figure 1. Core microbiome analysis in Hong Kong children. A. The relative abundance of 7 most prevalent genera. B. Heatmap showing the prevalence of abundant genera.

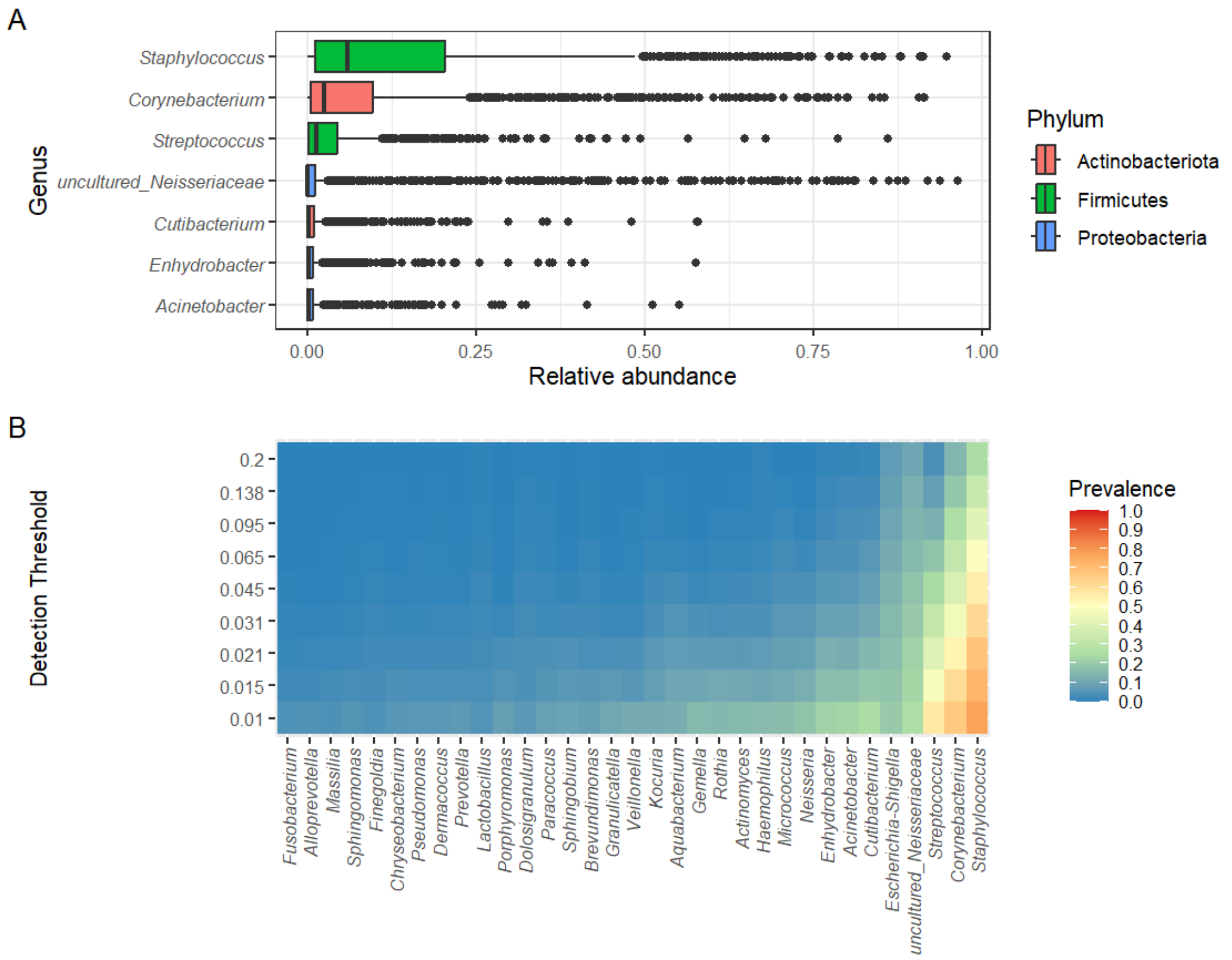

Supplementary Figure 2. **Analysis of OSM laterality in children.** A. Comparative average relative abundance of the ten most abundant genera in the right and left eyes. B. Similarity in Shannon and Simpson diversity indices between the left and right eyes. C. Principal coordinate analysis (PCoA) plot indicating no significant difference in microbial community composition between the left and right eyes based on Bray-Curtis distances. D. Bray-Curtis distances within paired samples from the same individual are significantly smaller compared to those from unpaired samples.

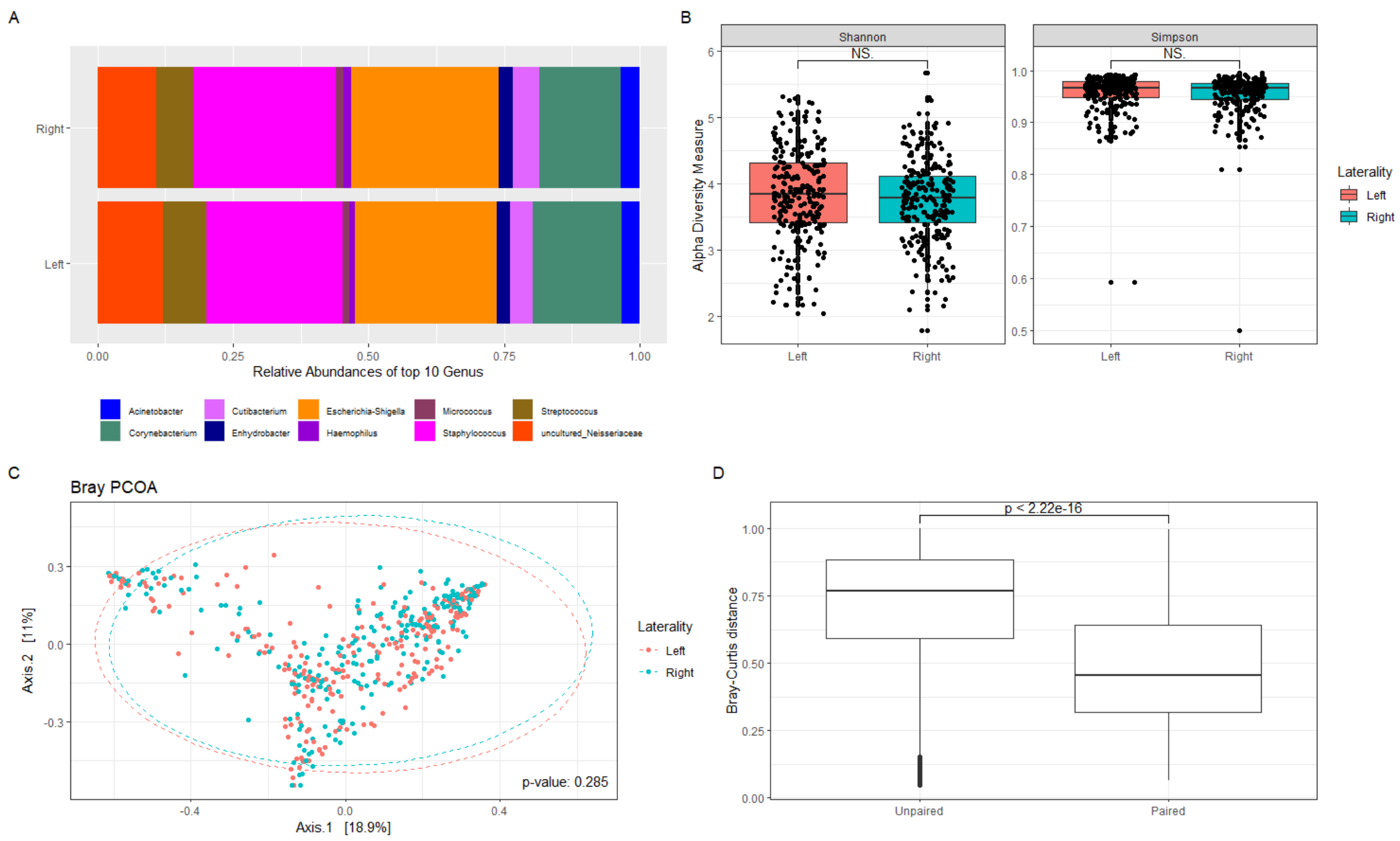

Supplementary Figure 3. Optimal number of clusters was identified as five by CH index within all samples.

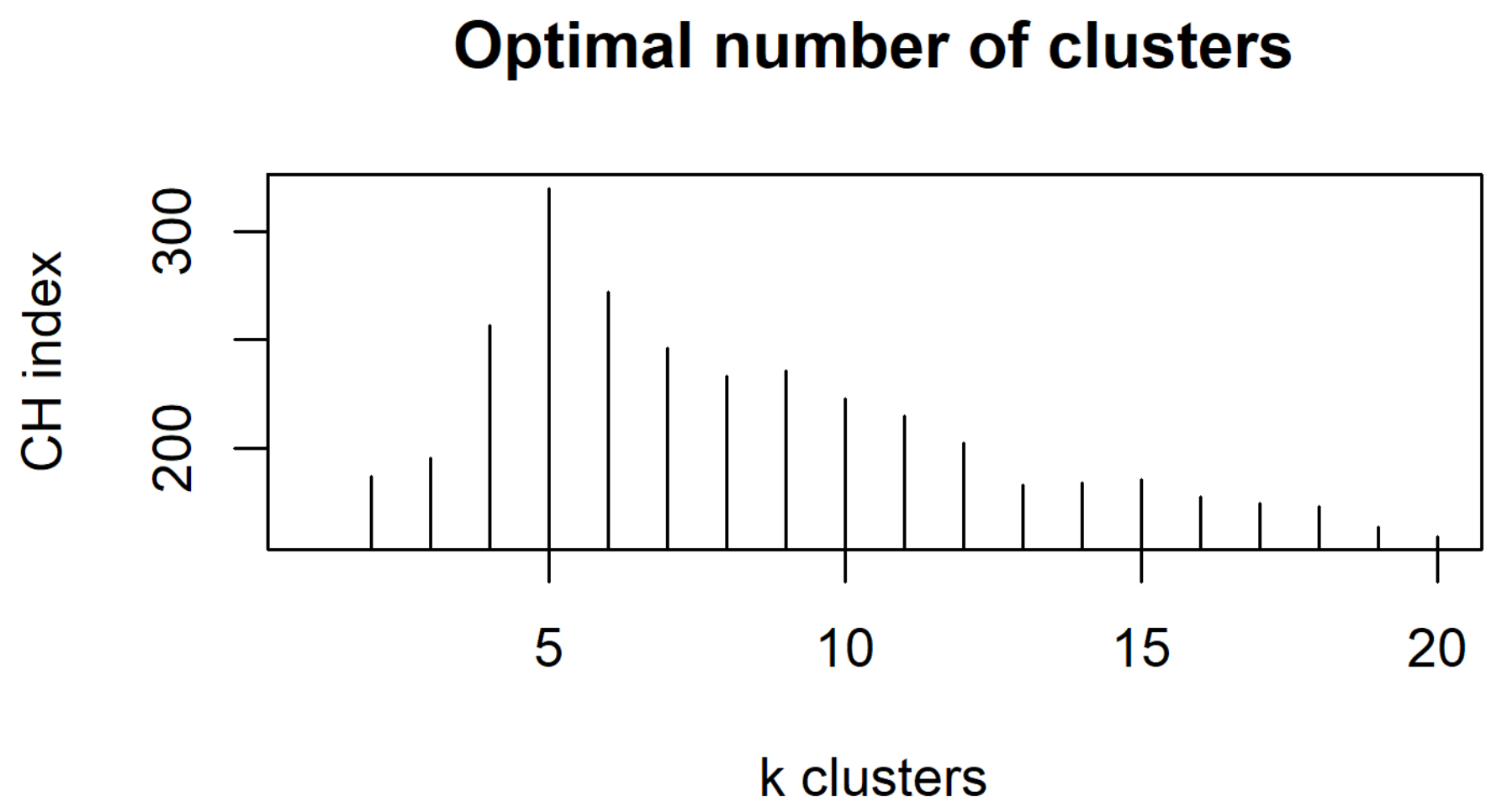

Supplementary Figure 4. Silhouette Index of each sample. This figure shows the Silhouette Index for each sample, serving as an evaluation of the clustering quality. Each line on the horizontal axis (x-axis) represents a unique sample, arranged according to cluster assignments. The corresponding Silhouette Index score for each sample is indicated on the vertical axis

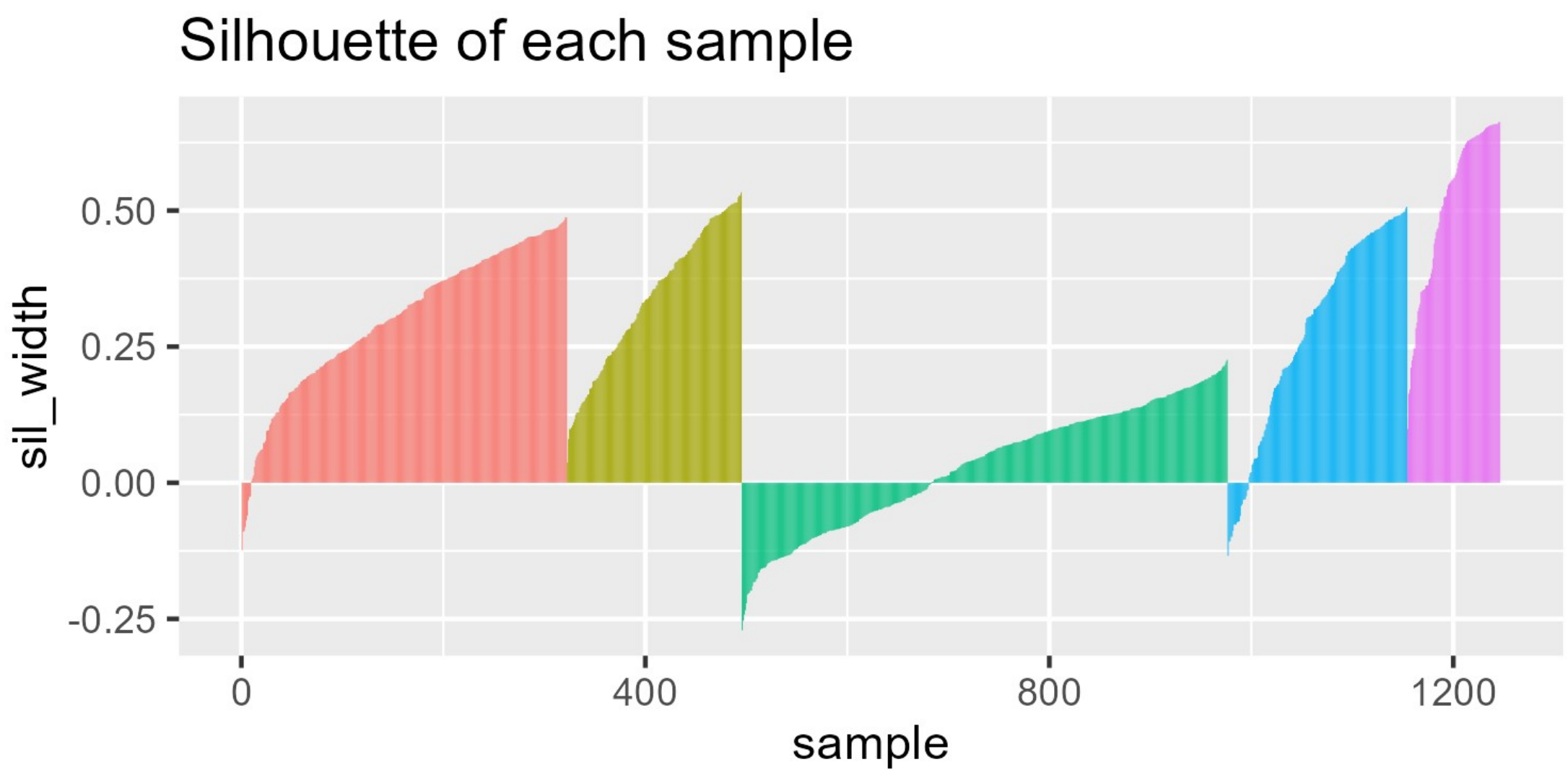

Supplementary Figure 5. Marker Genera Identification Across Ocular types. This bar plot visualizes the identification of distinctive marker genera within each ocular type, as determined by the LDA (Linear Discriminant Analysis) effect size (LDA score >4) through LEfSe (LDA Effect Size) analysis.

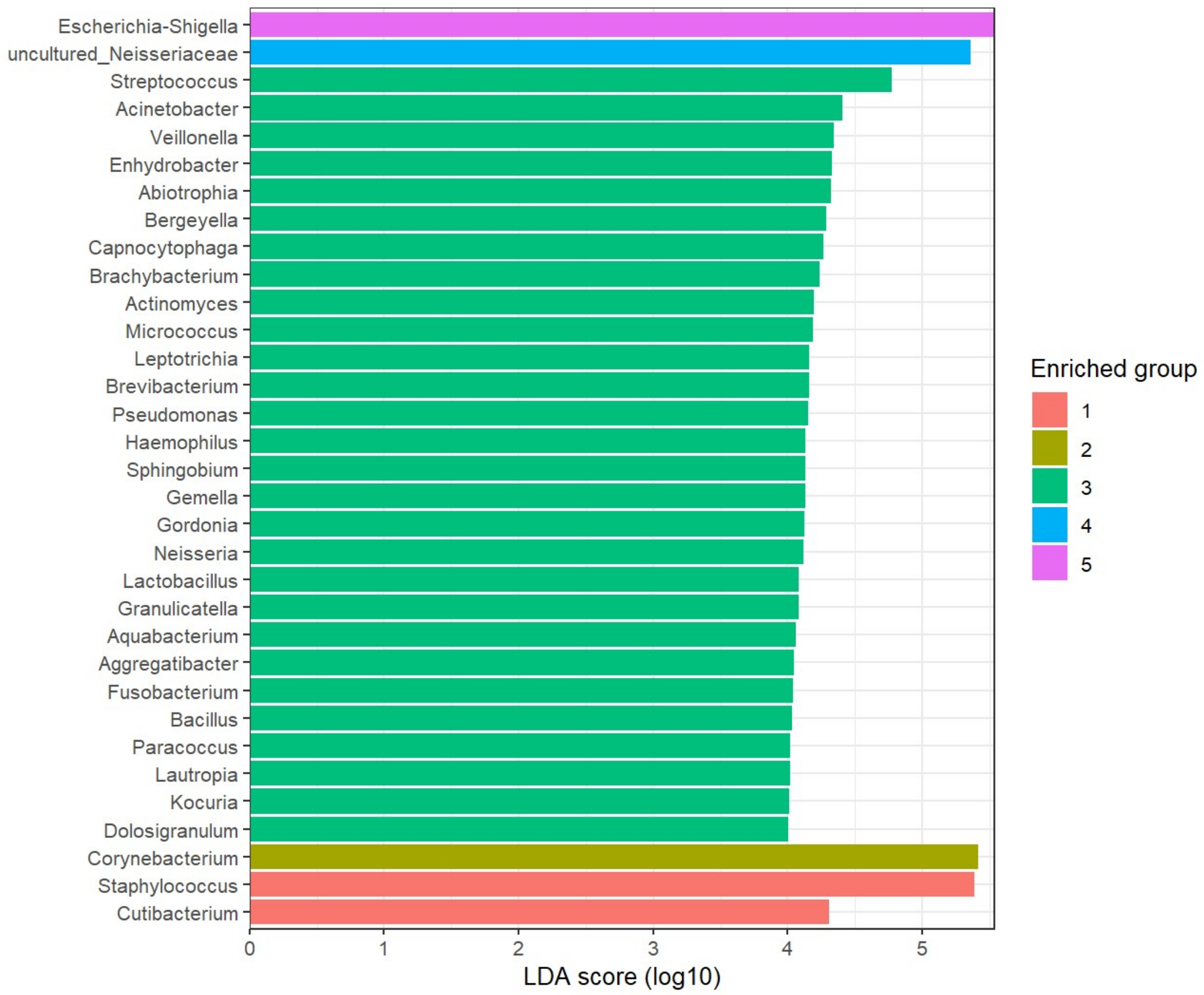

Supplementary Figure 6. Optimal number of clusters was identified as five by CH index within Subset 1 (60% samples).

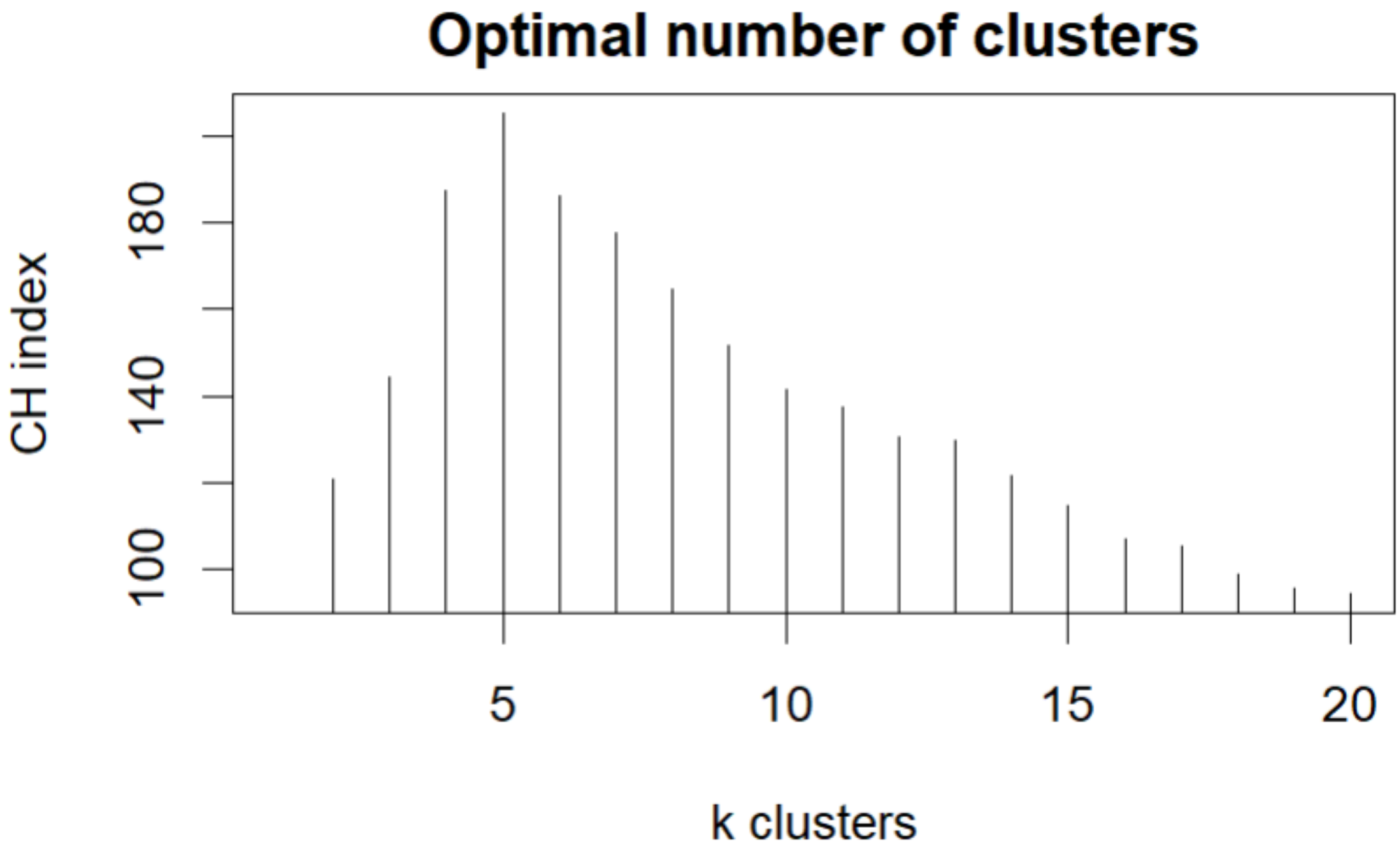

Supplementary Figure 7. BCA analysis on the five ocular types of Subset 1 (60% samples).

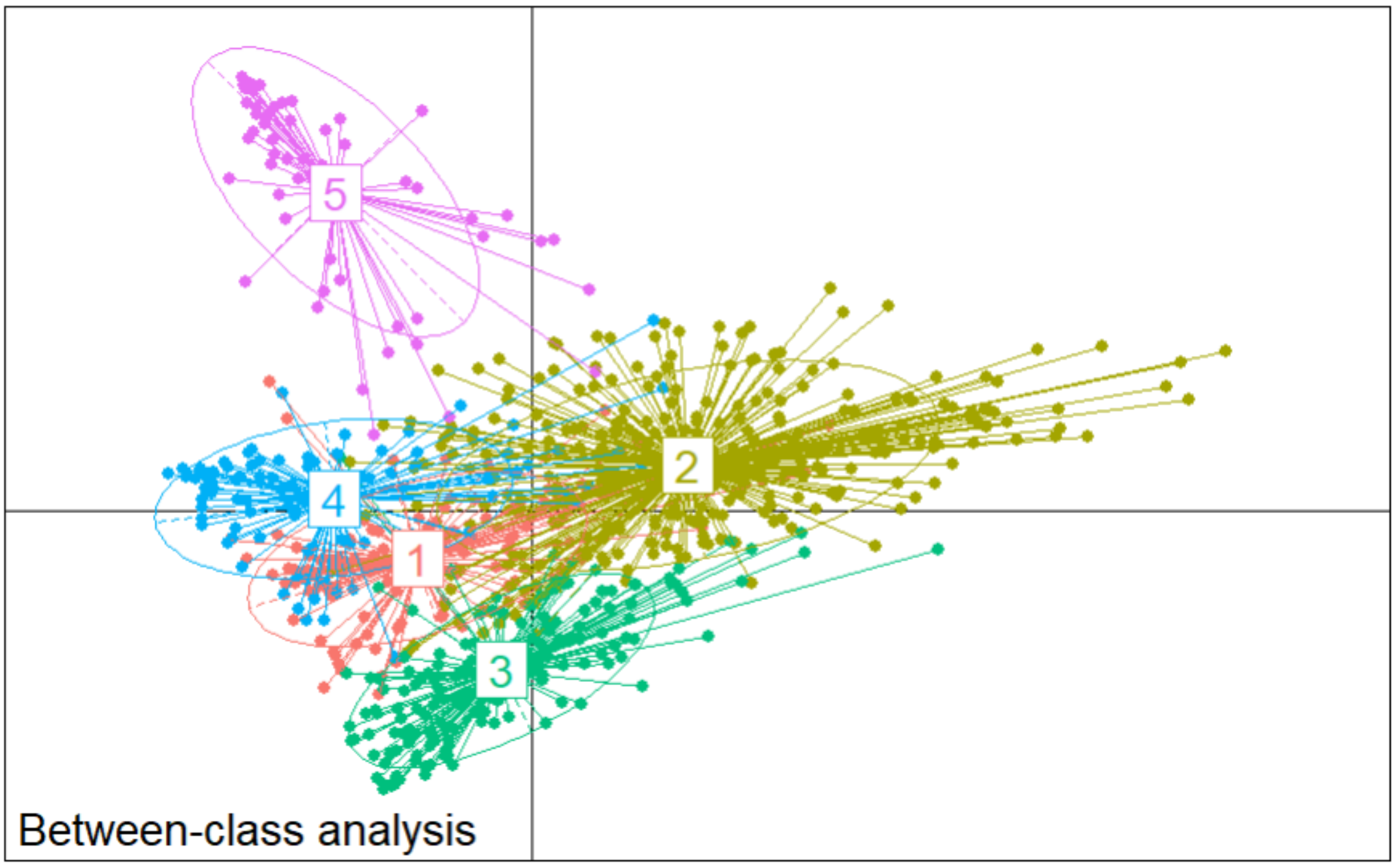

Supplementary Figure 8. PCoA analysis on the five ocular types of Subset 1 (60% samples).

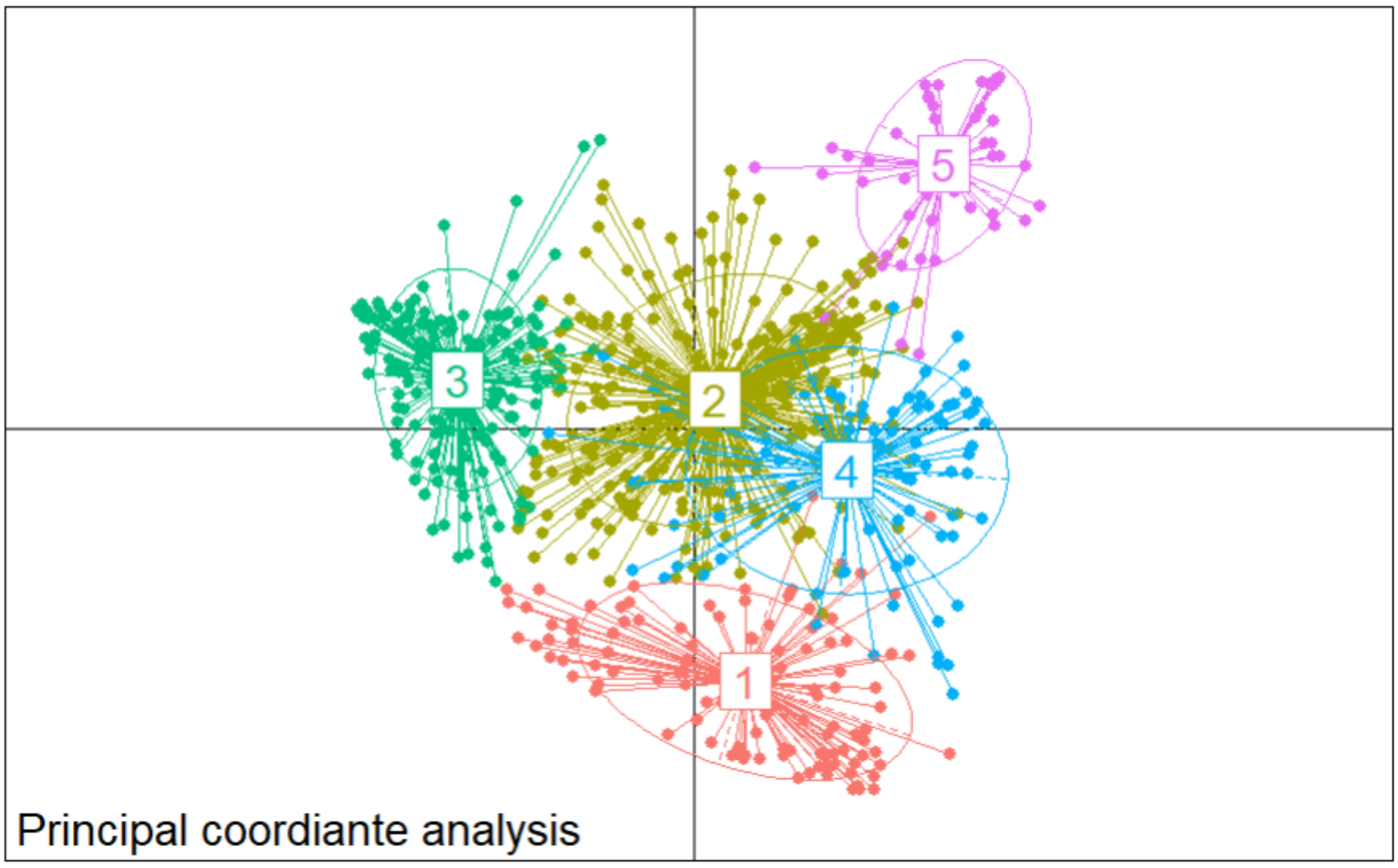

Supplementary Figure 9. The relative abundance of 5 representative genera in Subset 1 (60% samples).

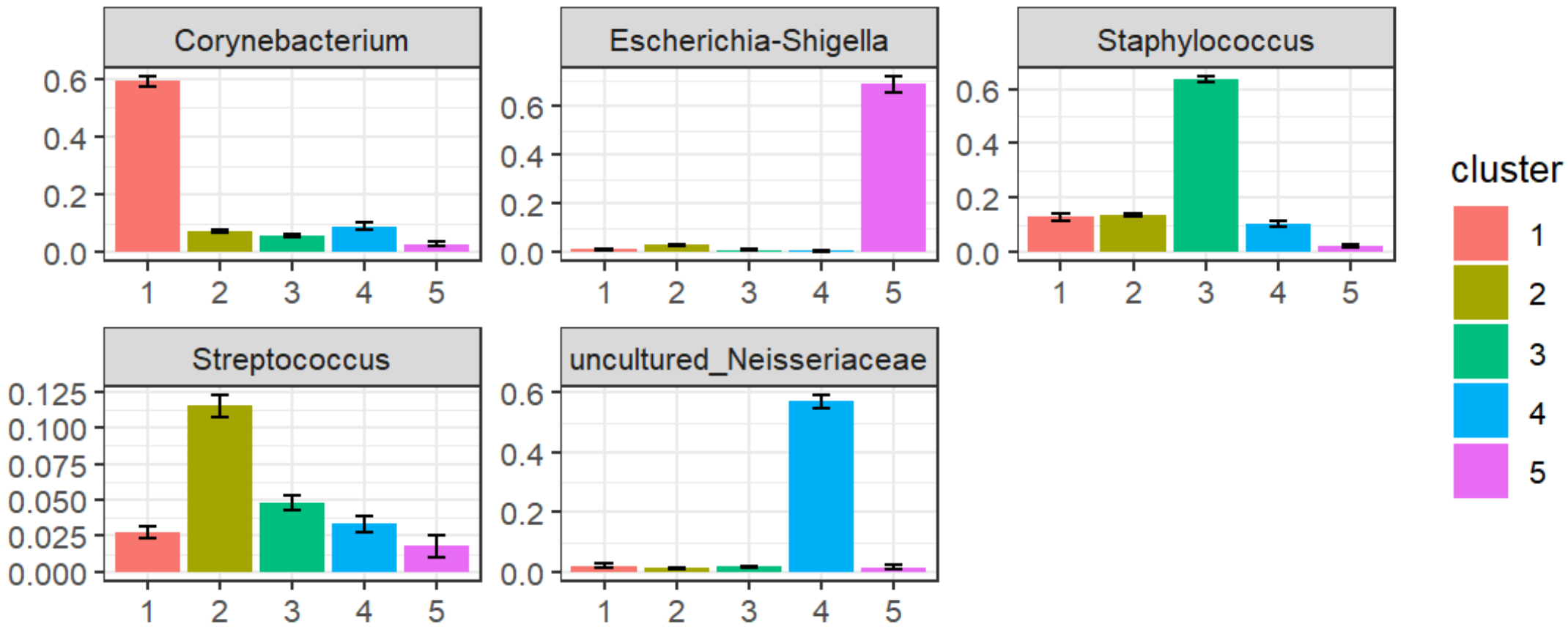

Supplementary Figure 10. Optimal number of clusters was identified as five by CH index within Subset 2 (40% samples).

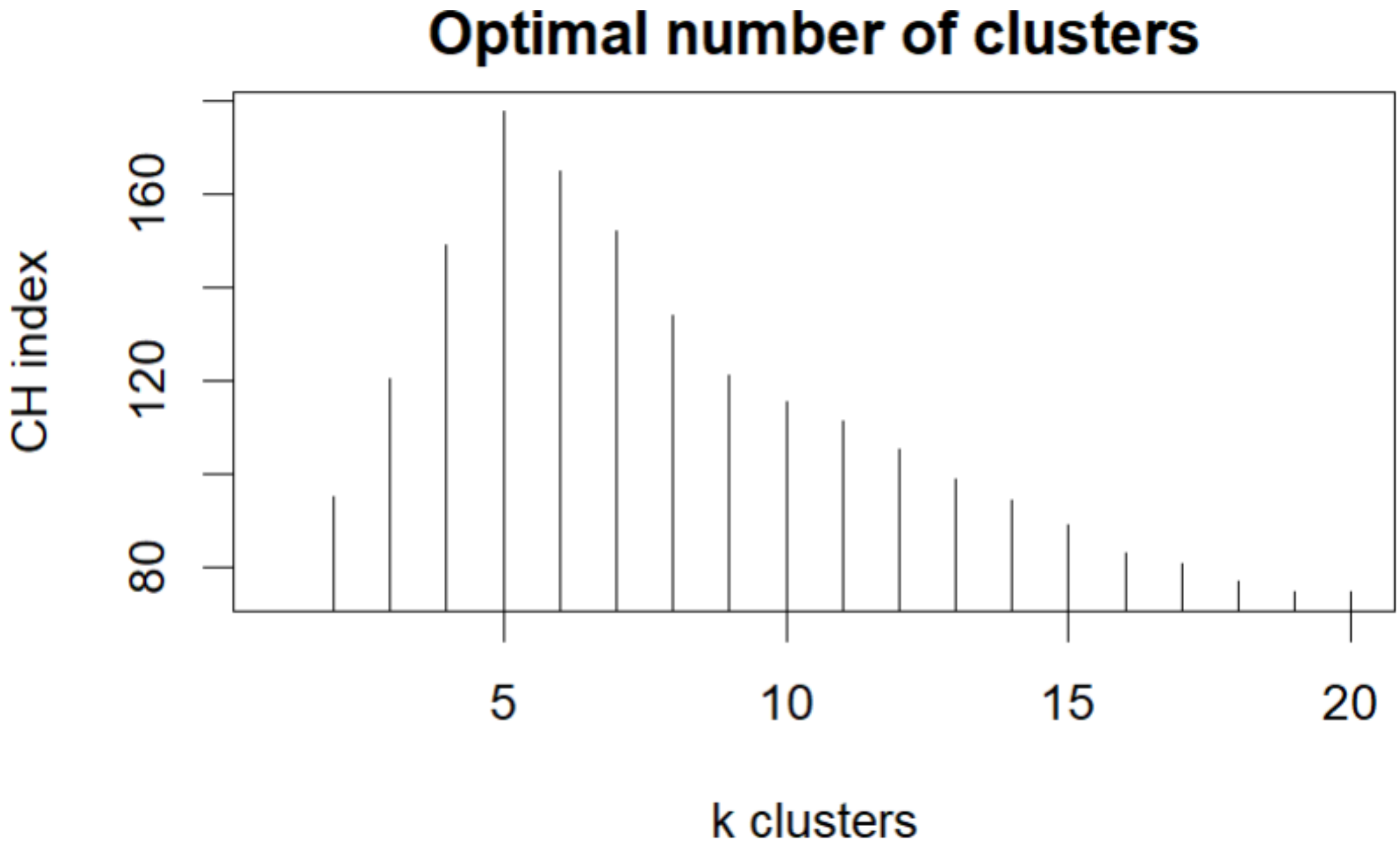

Supplementary Figure 11. BCA analysis on the five ocular types of Subset 2 (40% samples).

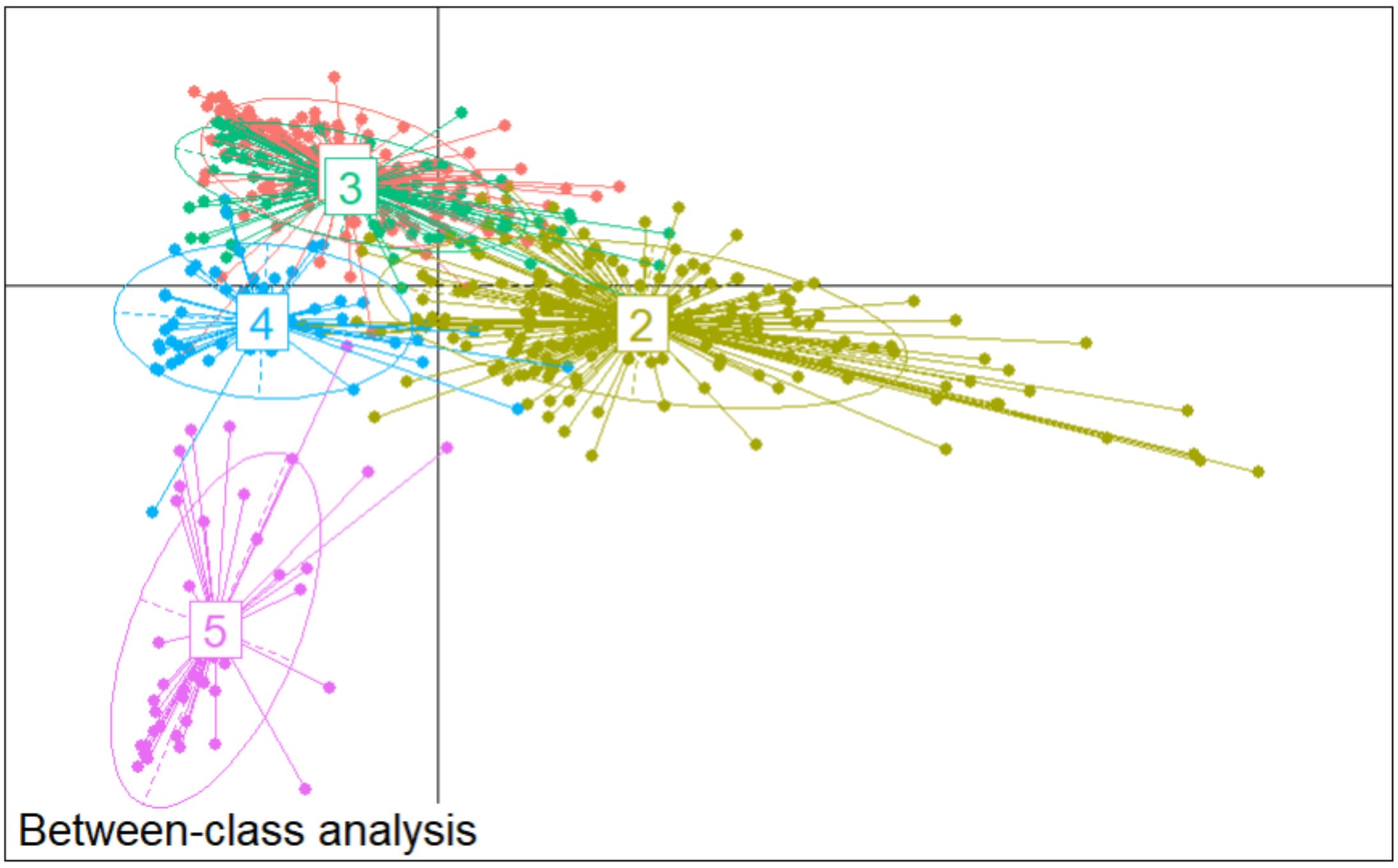

Supplementary Figure 12. PCoA analysis on the five ocular types of Subset 2 (40% samples).

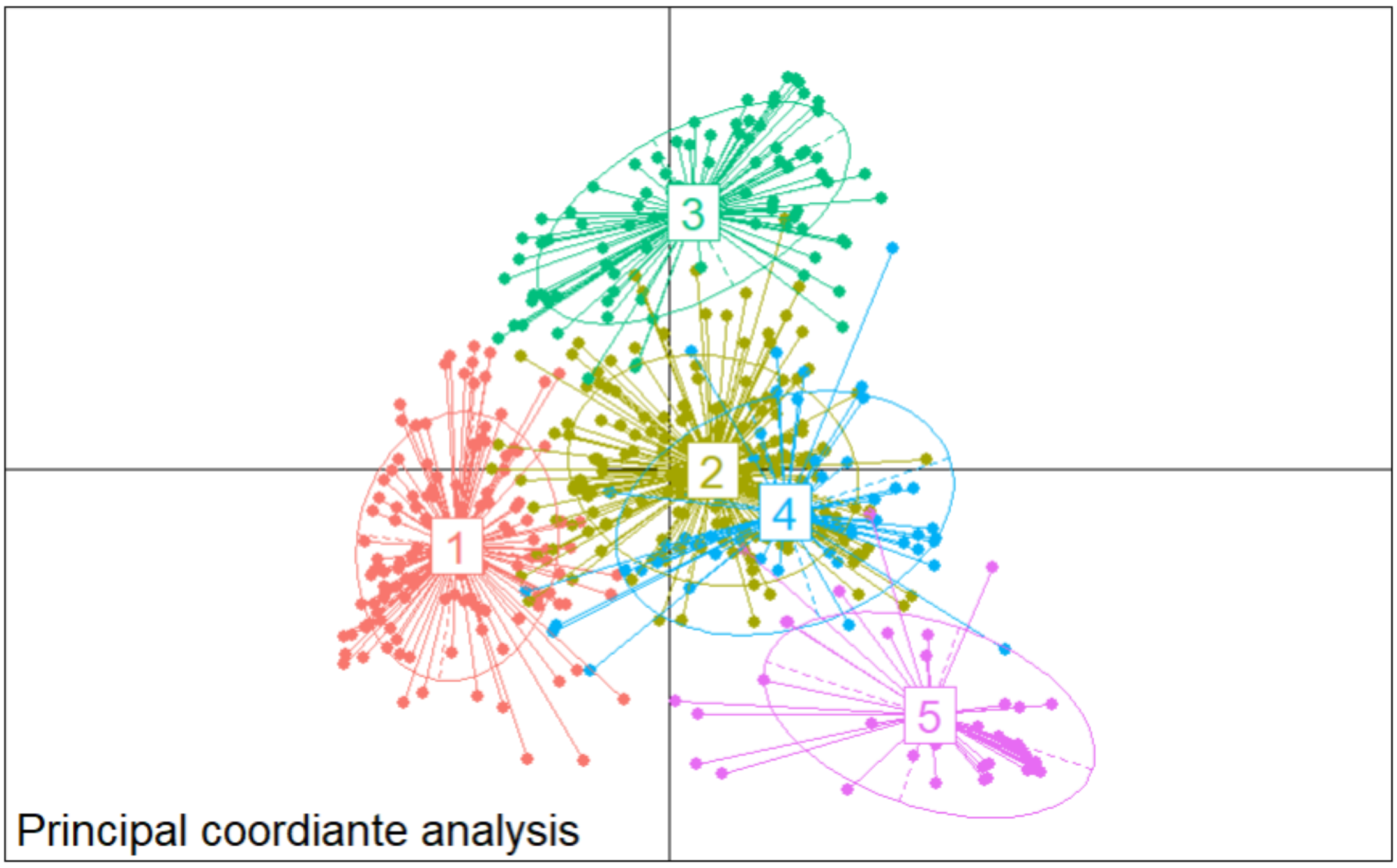

Supplementary Figure 13. The relative abundance of 5 representative genera in Subset 2 (40% samples).

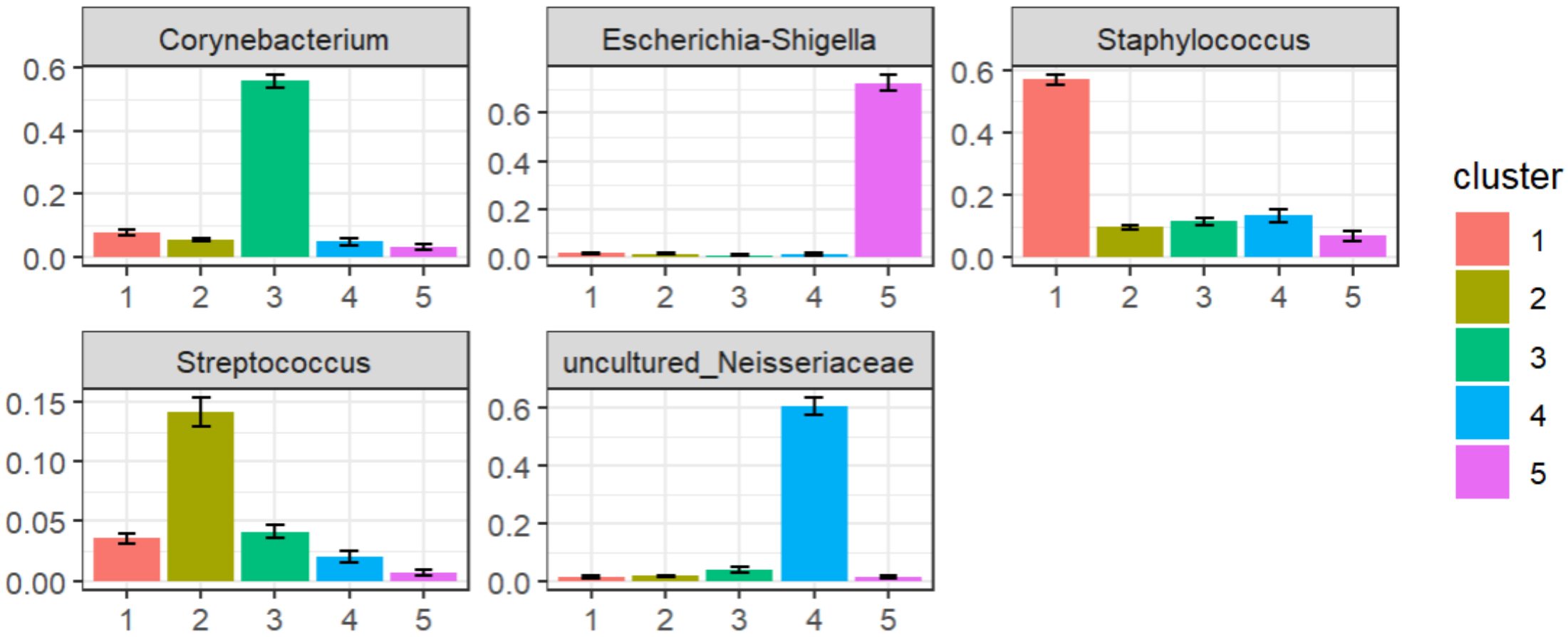

Supplementary Figure 14. Optimal number of clusters was identified as three by CH index within external Dataset 1 (Yau's study on allergic rhinoconjunctivitis).

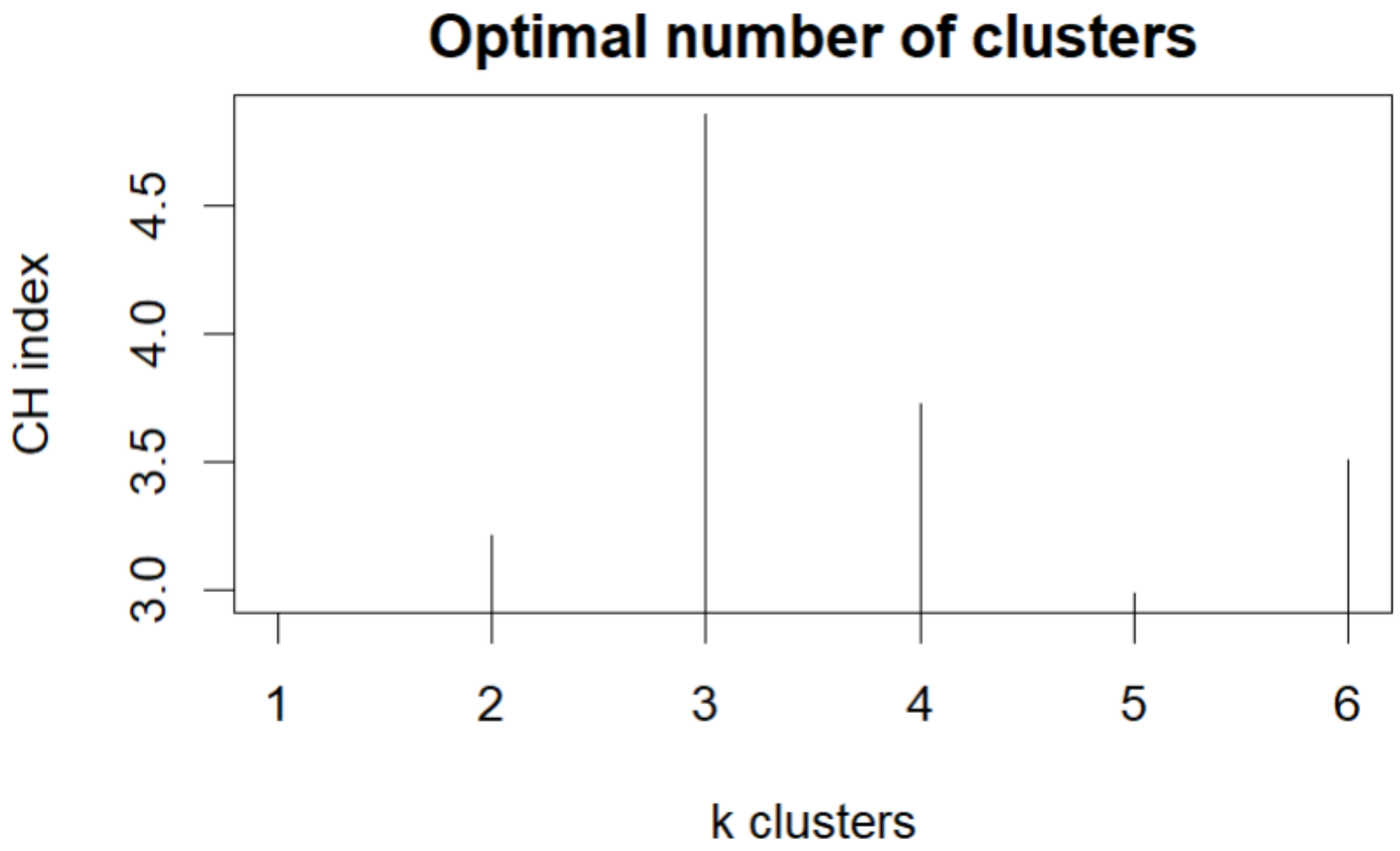

Supplementary Figure 15. BCA analysis on the five clusters of external Dataset 1 (Yau's study on allergic rhinoconjunctivitis).

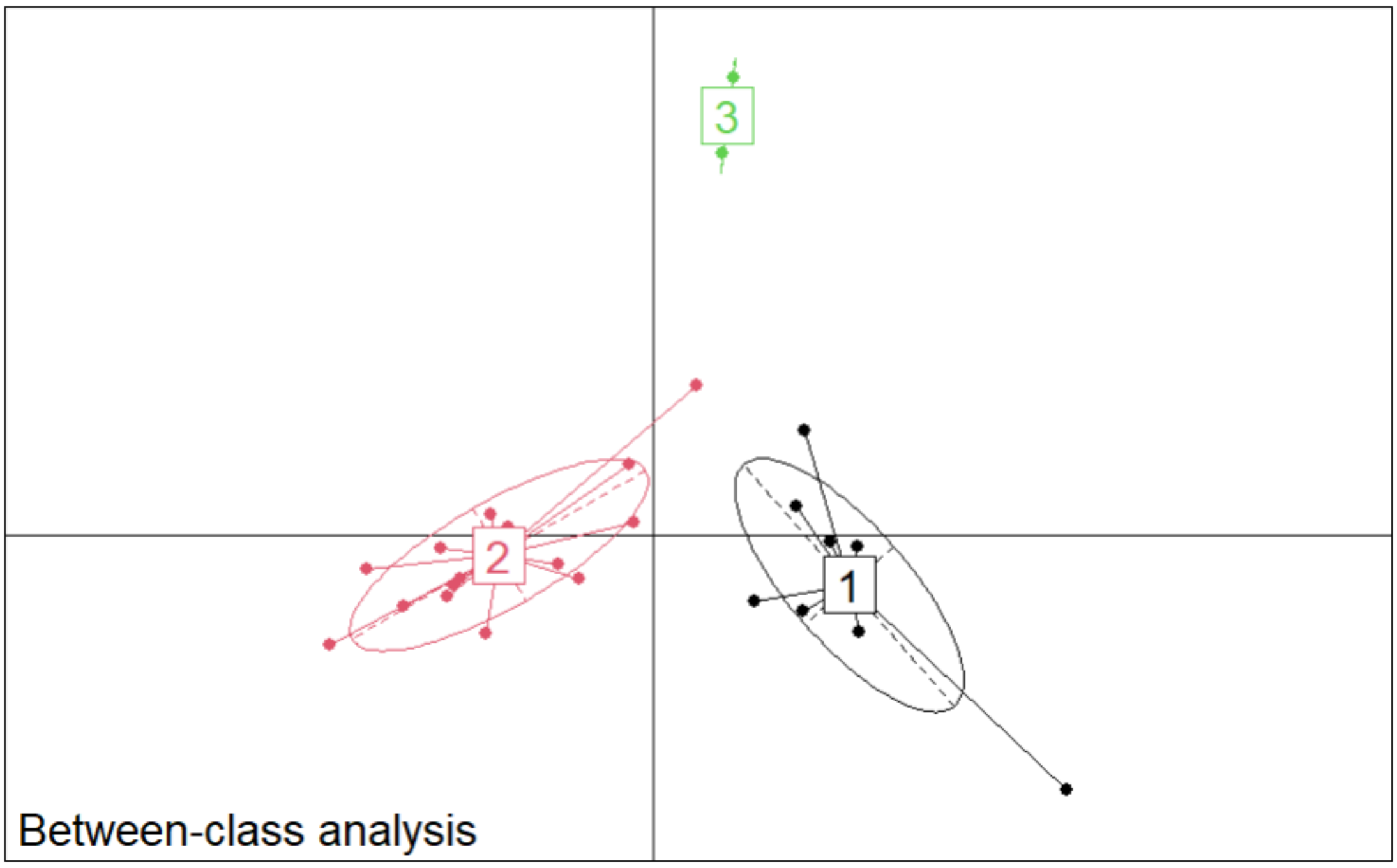

Supplementary Figure 16. The relative abundance of 3 representative genera in external Dataset 1 (Yau's study on allergic rhinoconjunctivitis).

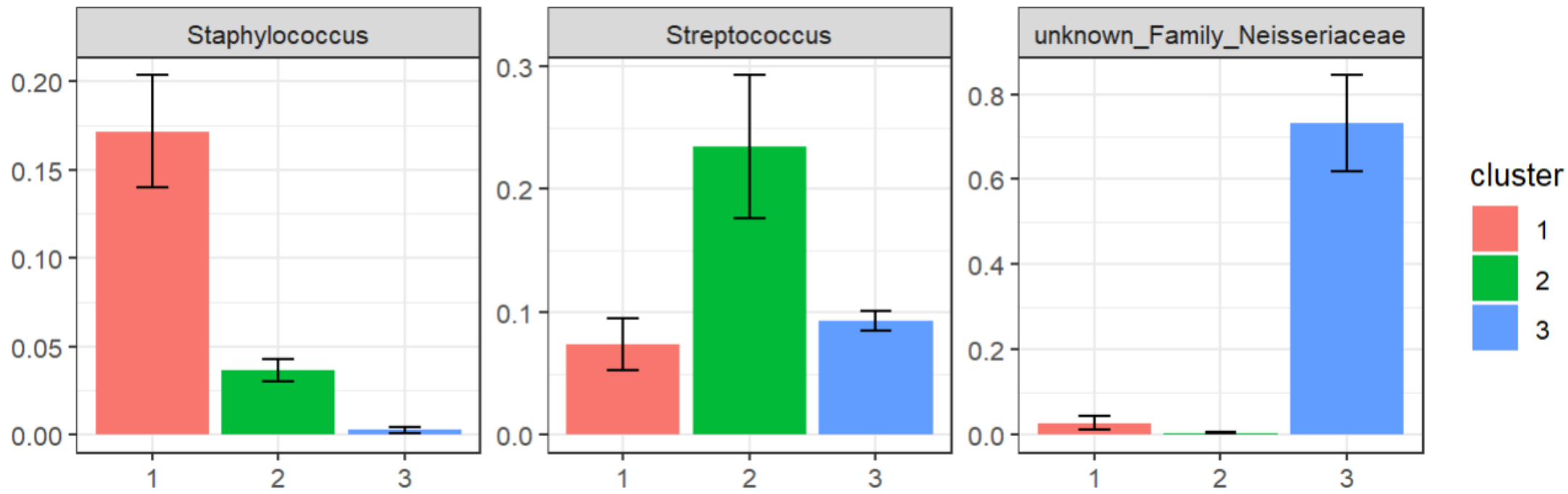

Supplementary Figure 17. Optimal number of clusters was identified as three by CH index within external Dataset 2 (Ozkan's study on dry eye diseases).

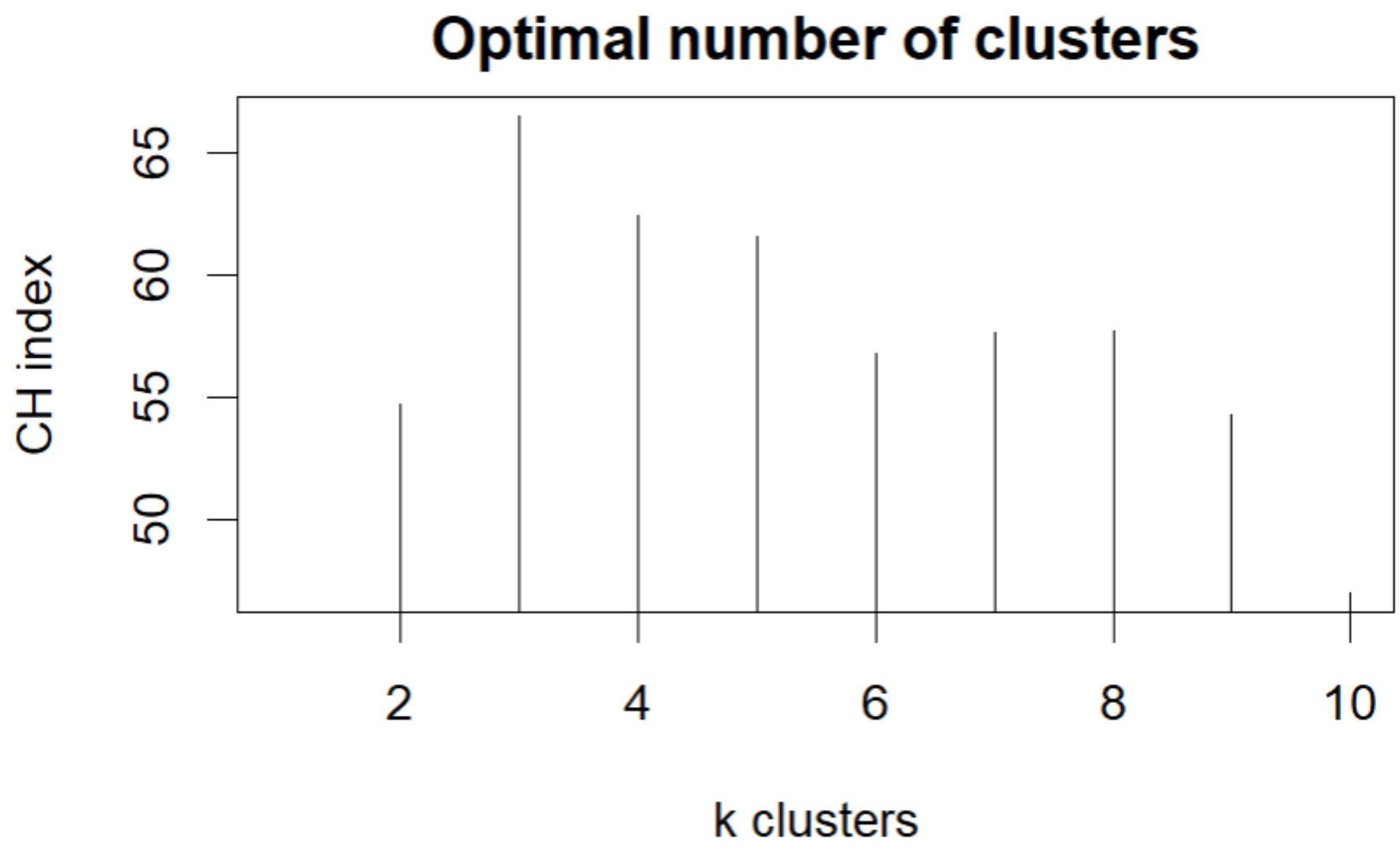

Supplementary Figure 18. BCA analysis on the five ocular types of external Dataset 2 (Ozkan's study on dry eye diseases).

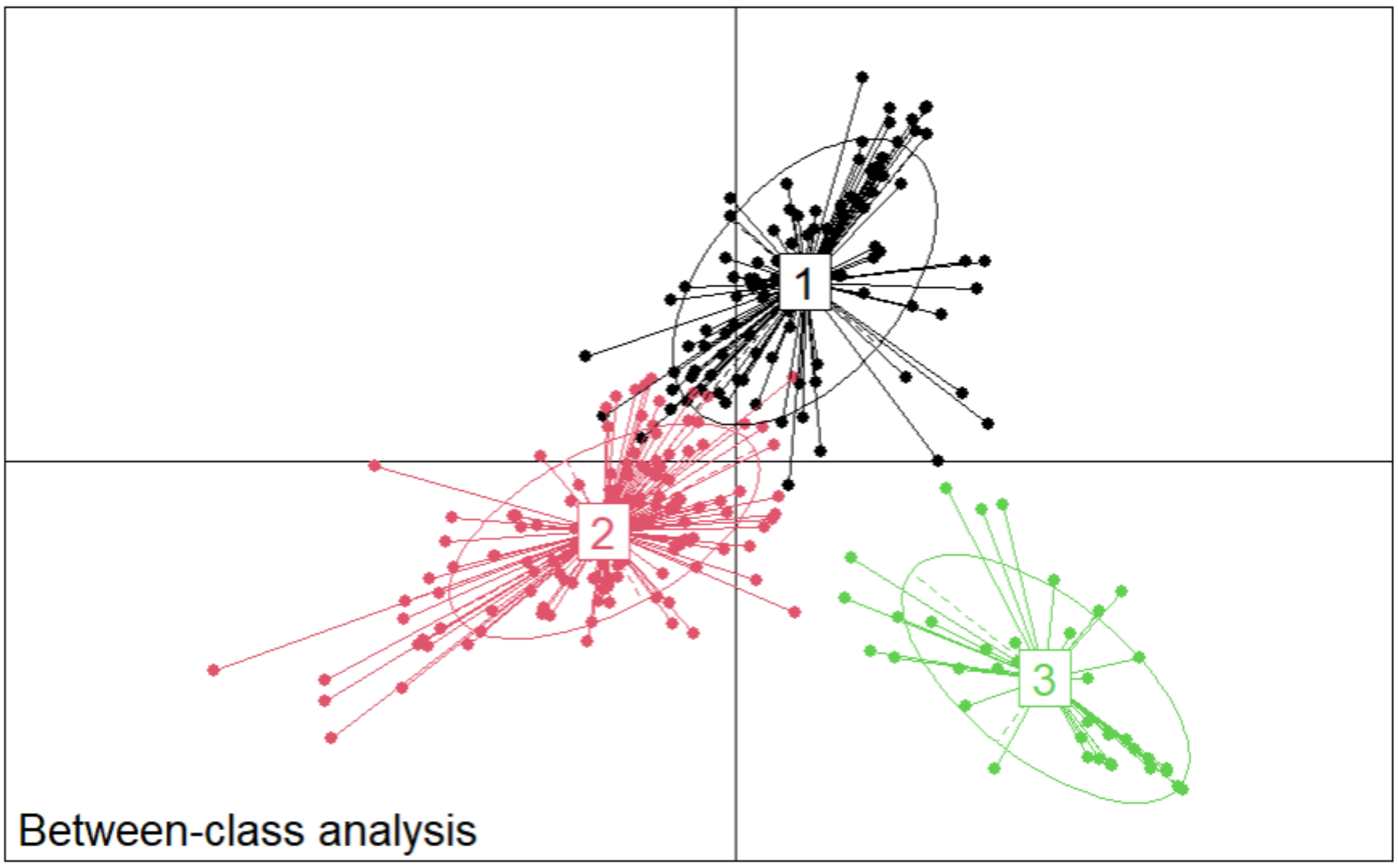

Supplementary Figure 19. The relative abundance of 3 representative genera in external Dataset 2 (Ozkan's study on dry eye diseases).

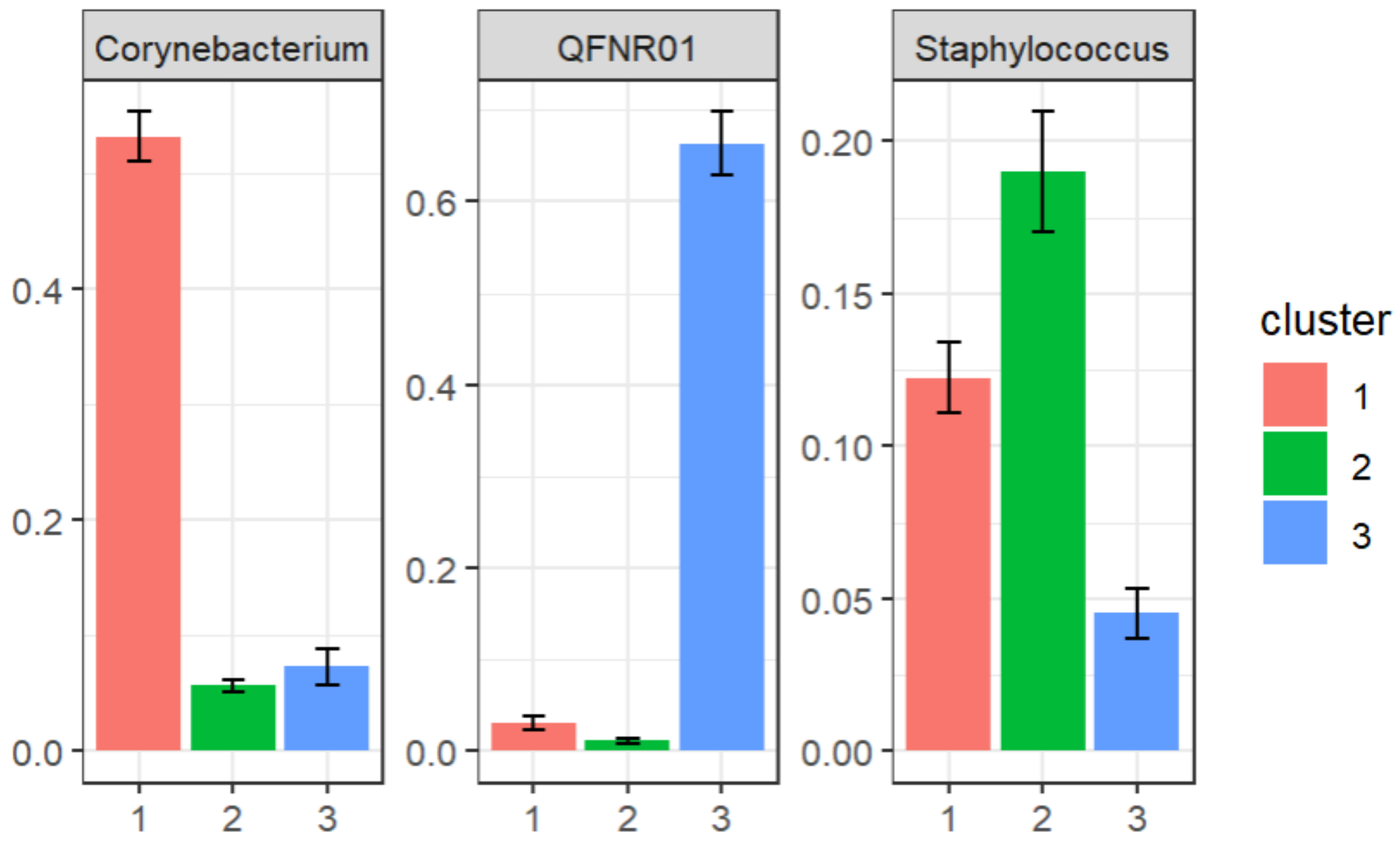

Supplementary Figure 20. Optimal number of clusters was identified as two by CH index within external Dataset 3 (Dong's study on meibomian gland dysfunction).

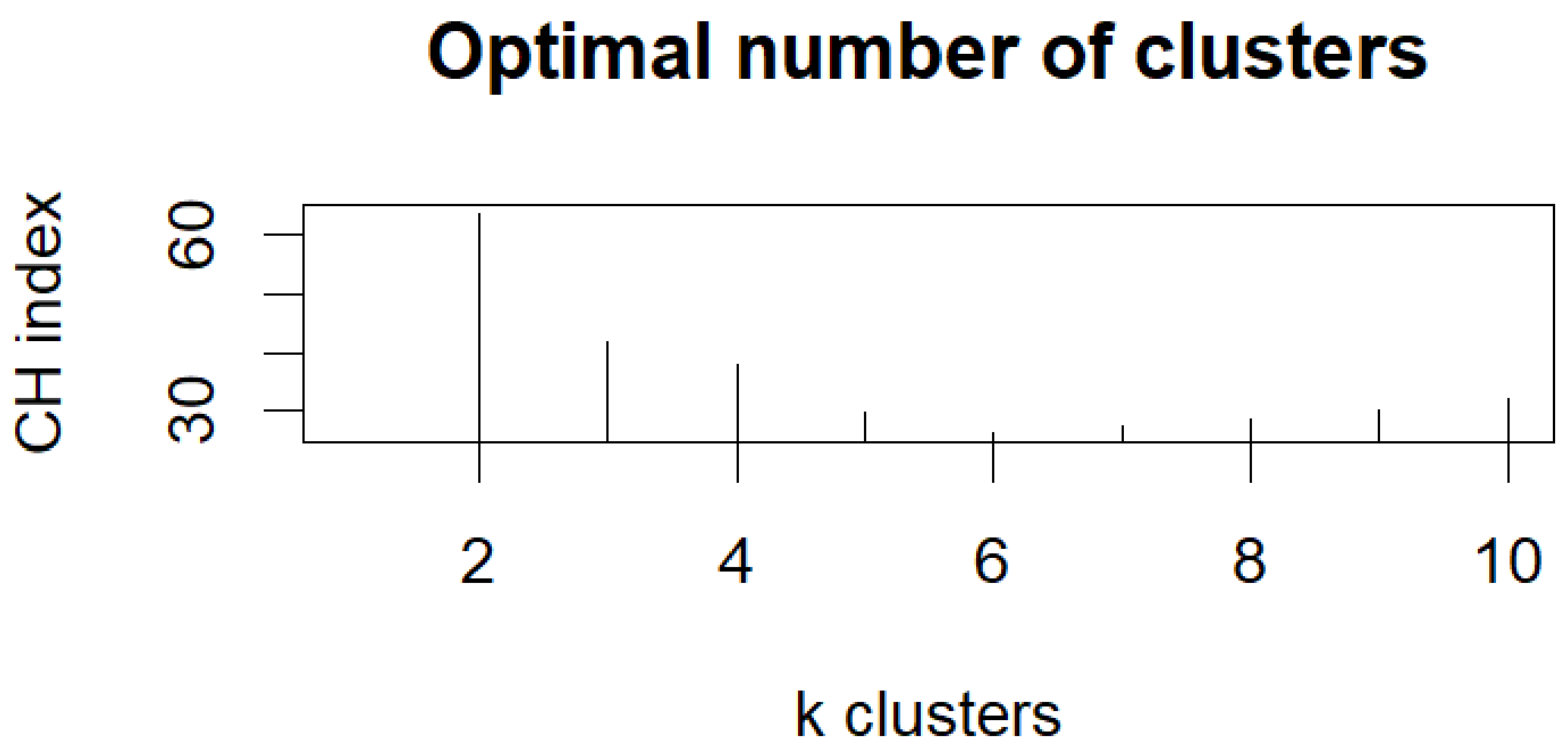

Supplementary Figure 21. The relative abundance of 2 representative genera in external Dataset 3 (Dong's study on meibomian gland dysfunction).

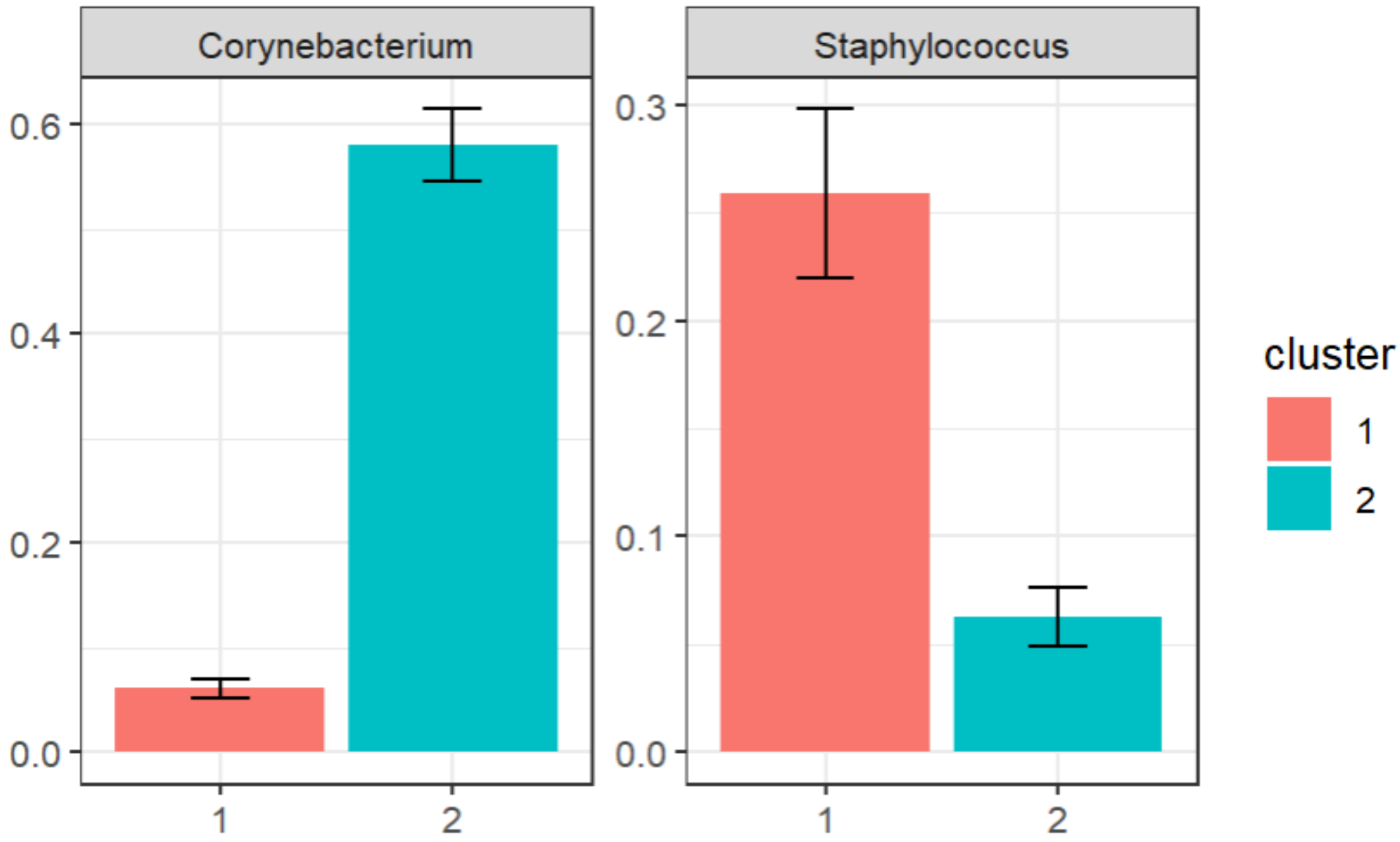

Supplementary Figure 22. Optimal number of clusters was identified as two by CH index within external Dataset 4 (Song's study on Sjogrens Syndrome).

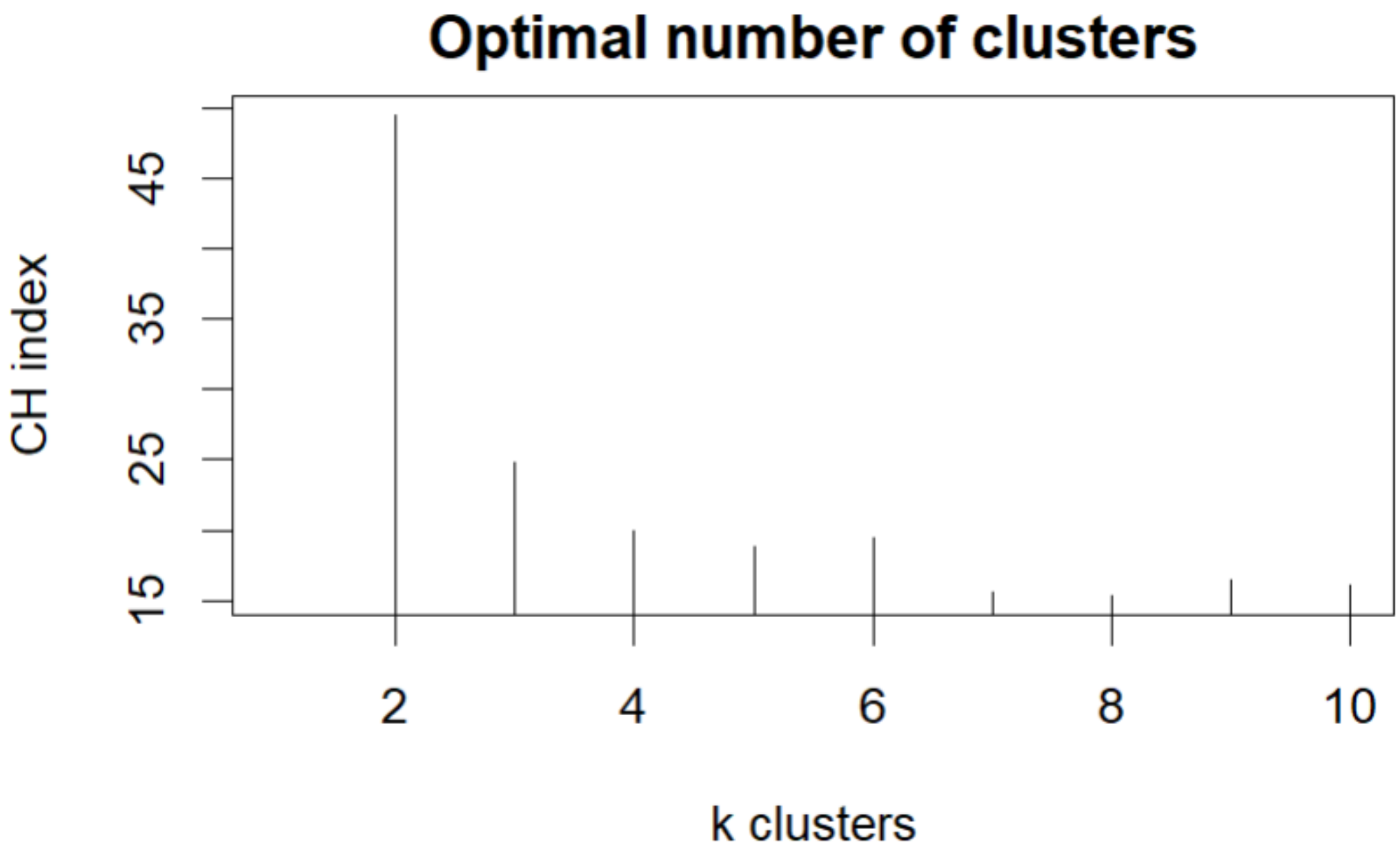

Supplementary Figure 23. The relative abundance of 2 representative genera in external Dataset 4 (Song's study on Sjogrens Syndrome).

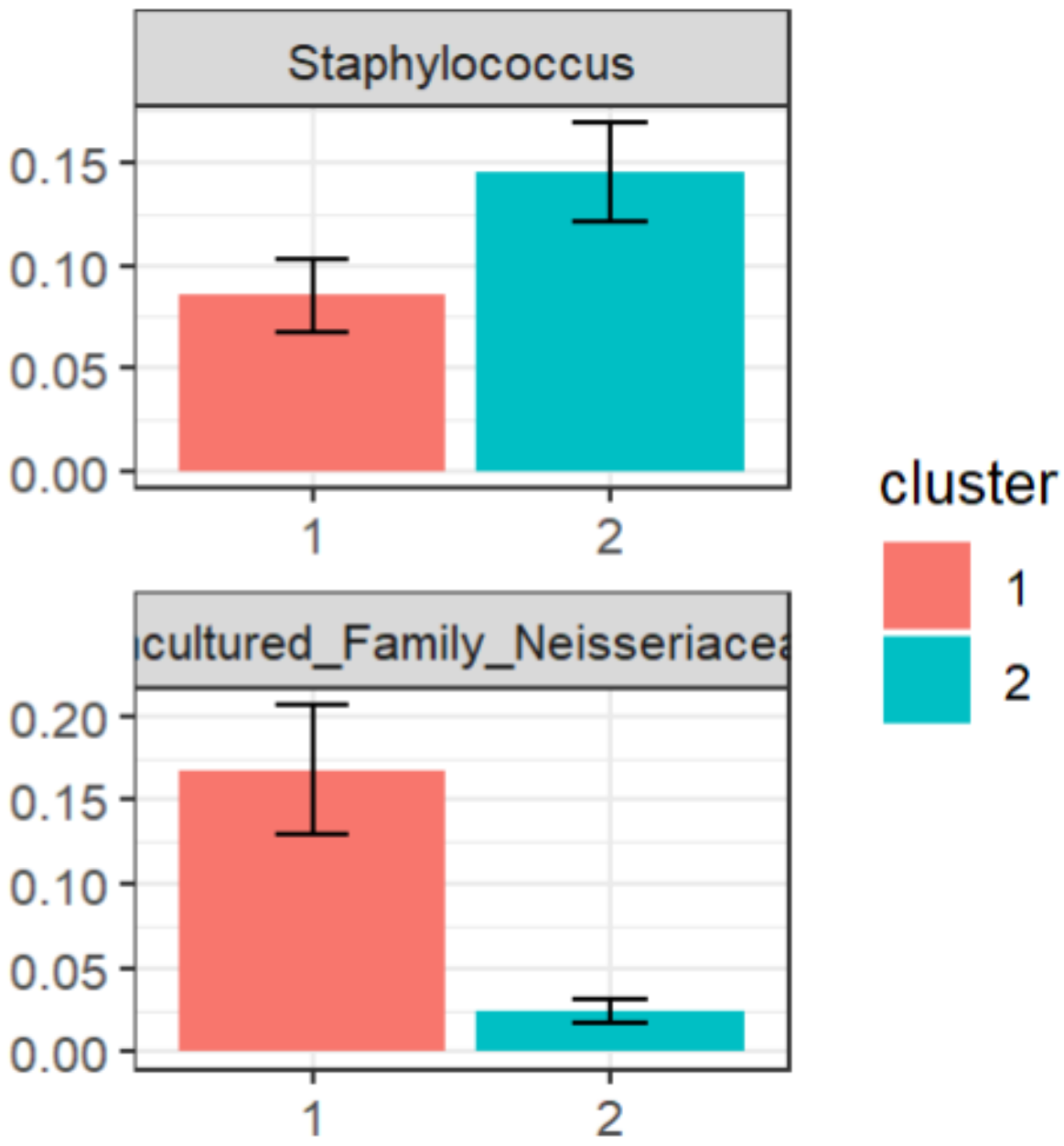

Supplementary Figure 24. Optimal number of clusters was identified as two by CH index within external Dataset 5 (Zhu's study on diabetes).

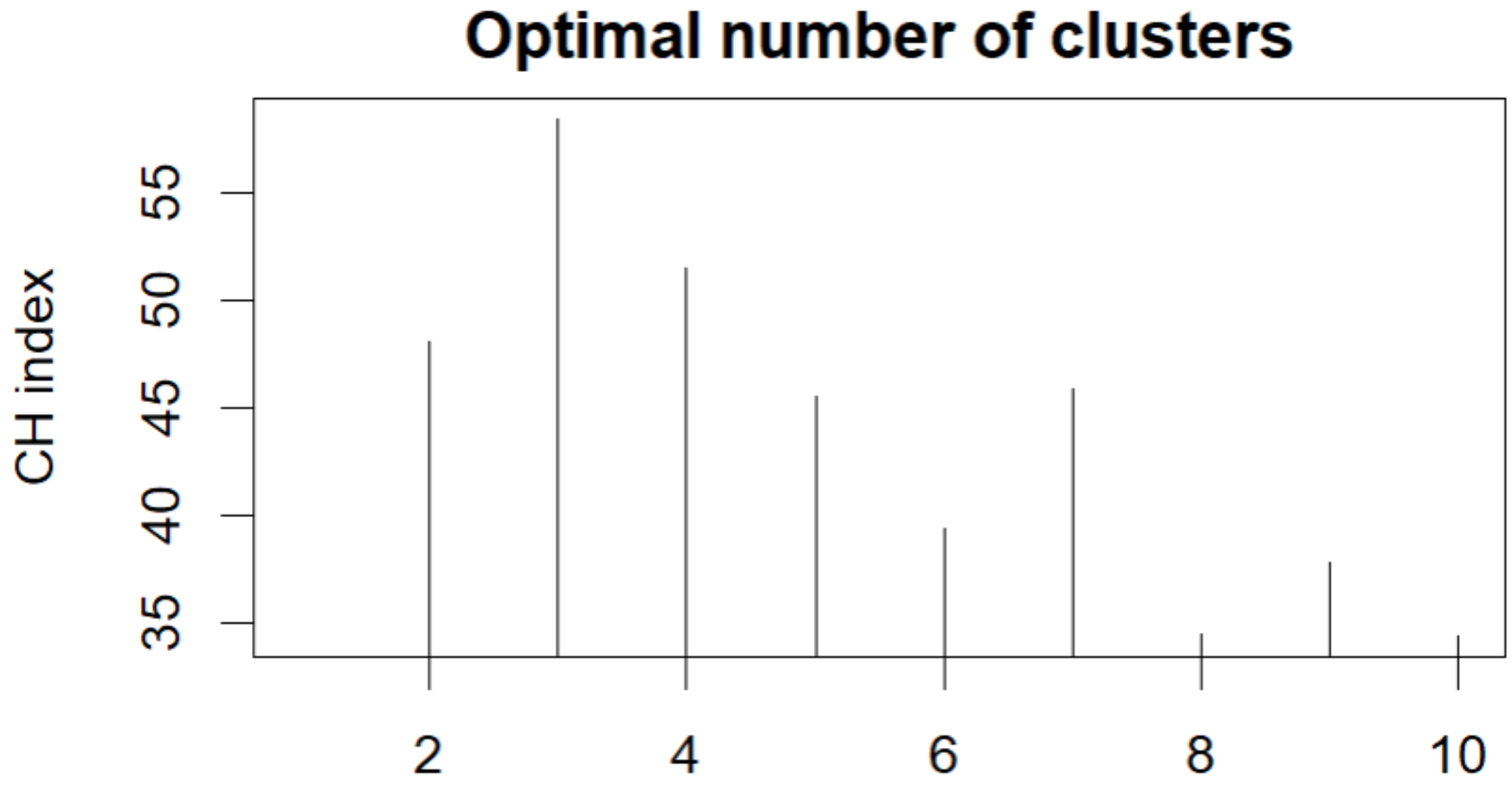

Supplementary Figure 25. The relative abundance of 3 representative genera in external Dataset 5 (Zhu's study on diabetes).

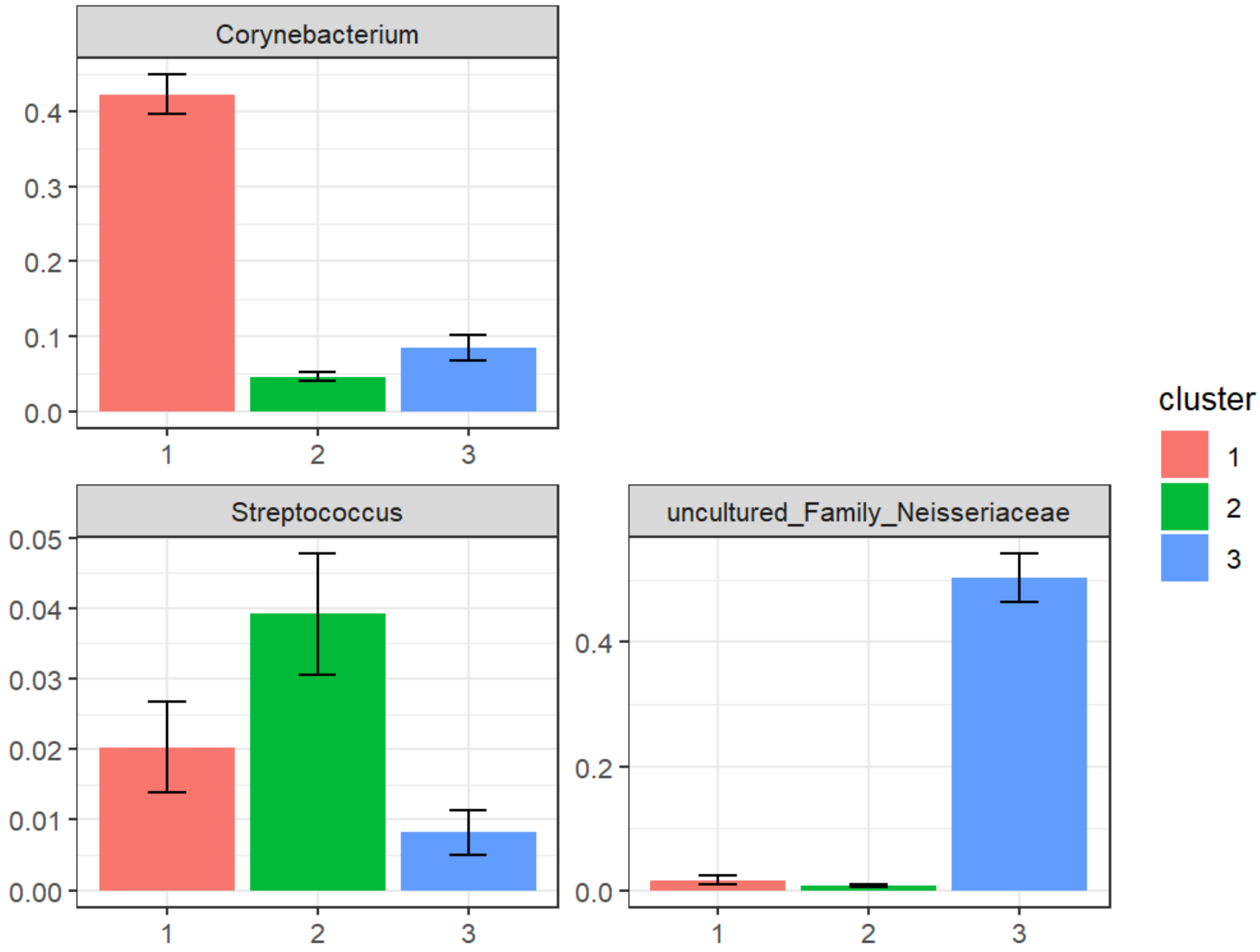

Supplementary Figure 26. LEfSe identified the different MetaCyc pathways among ocular types are presented.

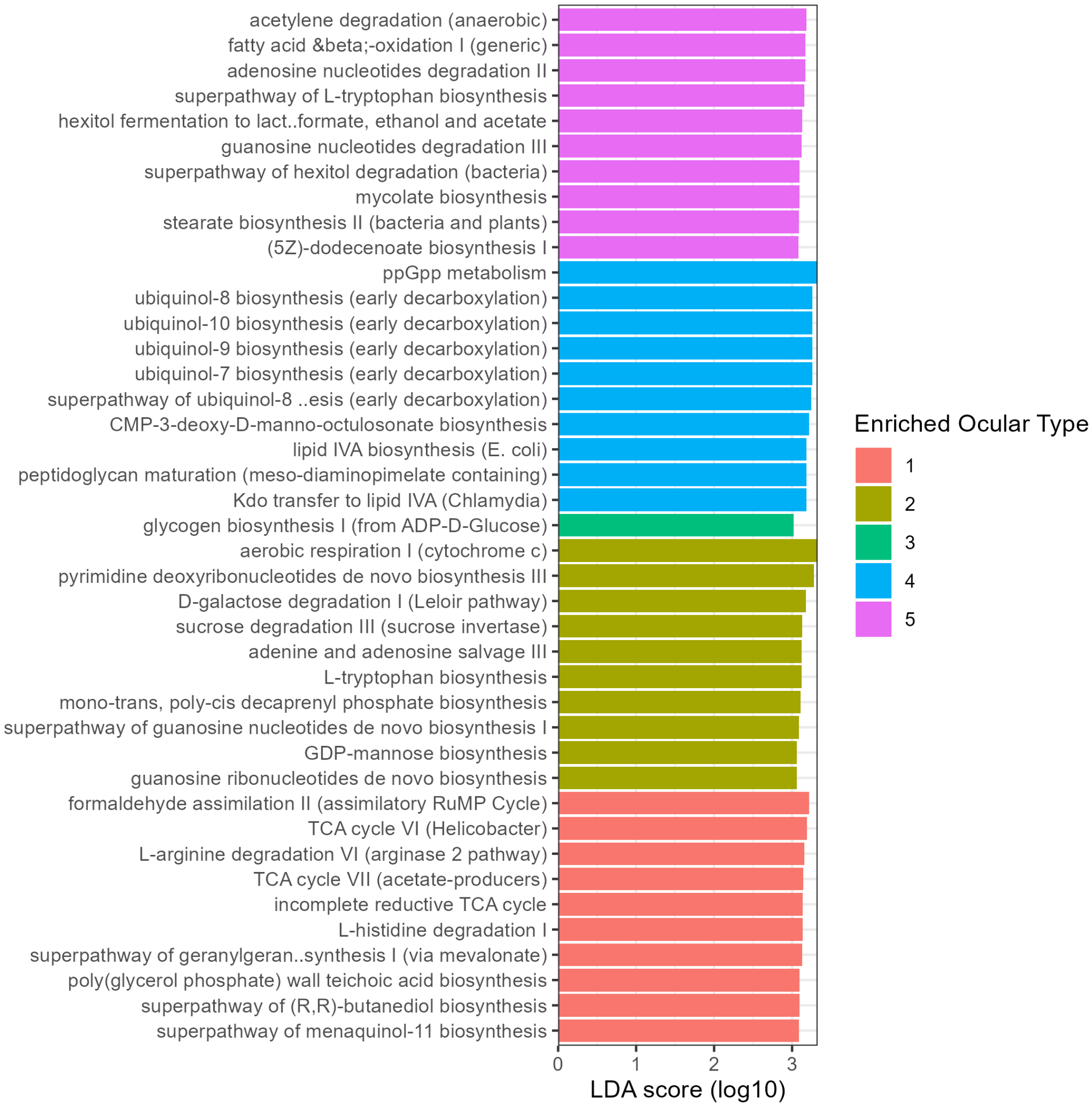

Supplementary Figure 27. Density chart showing the proportion of optimal cluster numbers under 1,000 repeated randomizations at each specific sample size.

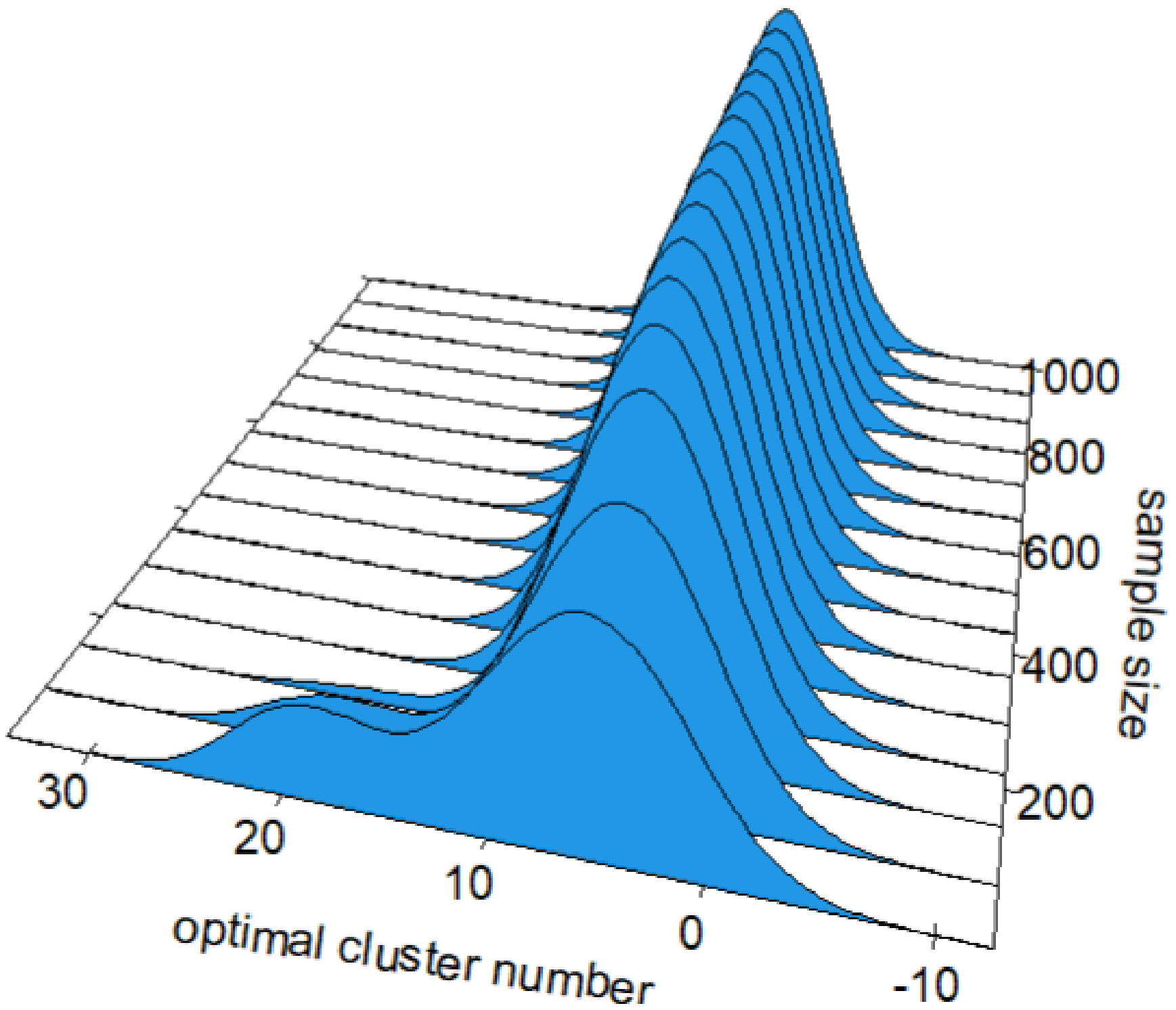

Supplementary Figure 28. Bar chart showing the proportion of optimal cluster numbers under 1,000 repeated randomizations at each specific sample size.

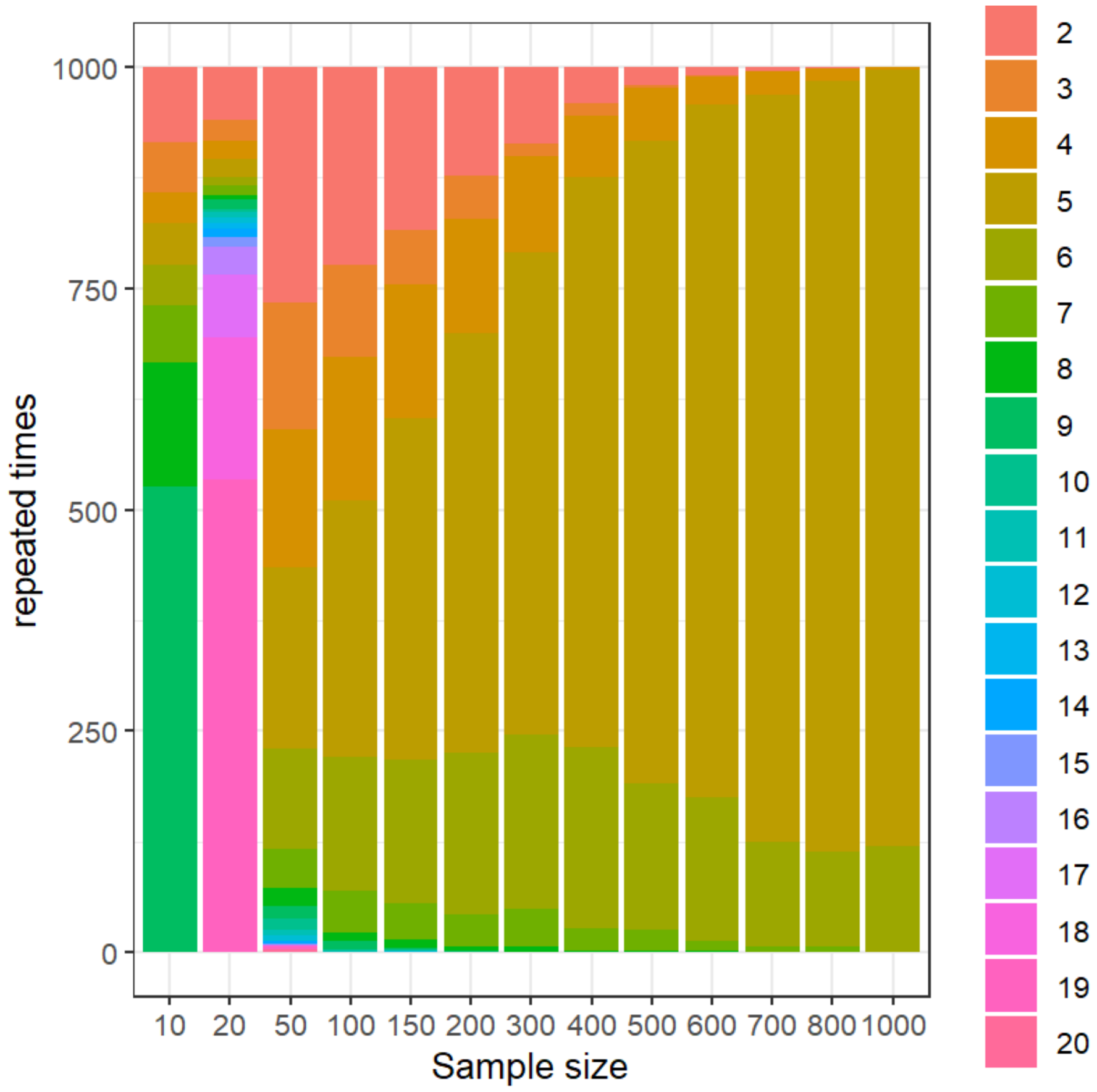

Supplement: Supplementary file 2 [file 43856_2026_1667_MOESM2_ESM.pdf]
